# A functionally conserved Zn_2_Cys_6_ binuclear cluster transcription factor class regulates necrotrophic effector gene expression and host‐specific virulence of two major Pleosporales fungal pathogens of wheat

**DOI:** 10.1111/mpp.12511

**Published:** 2017-01-24

**Authors:** Kasia Rybak, Pao Theen See, Huyen T. T. Phan, Robert A. Syme, Caroline S. Moffat, Richard P. Oliver, Kar‐Chun Tan

**Affiliations:** ^1^ Department of Environment & Agriculture, Centre for Crop and Disease Management Curtin University, Bentley, 6102 Perth Australia

**Keywords:** effector regulation, *Parastagonospora nodorum*, *Pyrenophora tritici‐repentis*, Septoria nodorum blotch, SnTox3, tan spot, ToxA

## Abstract

The fungus *Parastagonospora nodorum* is the causal agent of Septoria nodorum blotch of wheat (*Triticum aestivum*). The interaction is mediated by multiple fungal necrotrophic effector–dominant host sensitivity gene interactions. The three best‐characterized effector–sensitivity gene systems are SnToxA–*Tsn1*, SnTox1–*Snn1* and SnTox3–*Snn3*. These effector genes are highly expressed during early infection, but expression decreases as the infection progresses to tissue necrosis and sporulation. However, the mechanism of regulation is unknown. We have identified and functionally characterized a gene, referred to as *PnPf2*, which encodes a putative zinc finger transcription factor. *PnPf2* deletion resulted in the down‐regulation of *SnToxA* and *SnTox3* expression. Virulence on *Tsn1* and *Snn3* wheat cultivars was strongly reduced. The SnTox1–*Snn1* interaction remained unaffected. Furthermore, we have also identified and deleted an orthologous *PtrPf2* from the tan spot fungus *Pyrenophora tritici‐repentis* which possesses a near‐identical *ToxA* that was acquired from *P. nodorum* via horizontal gene transfer. *PtrPf2* deletion also resulted in the down‐regulation of *PtrToxA* expression and a near‐complete loss of virulence on *Tsn1* wheat. We have demonstrated, for the first time, evidence for a functionally conserved signalling component that plays a role in the regulation of a common/horizontally transferred effector found in two major fungal pathogens of wheat.

## Introduction

*Parastagonospora* (syn. *Stagonospora*, *Phaeosphaeria*, *Septoria*) *nodorum* (Berk.) Quaedvlieg, Verkley & Crous is the causal agent of Septoria nodorum blotch (SNB) of wheat (Quaedvlieg *et al*., 2013; Solomon *et al*., 2006a). The fungus causes significant damage to the leaves and glumes of wheat, and is responsible for substantial yield losses in many wheat‐growing areas (Eyal, 1999; Murray and Brennan, 2009; Oliver *et al*., 2014, 2016). *Parastagonospora nodorum* belongs to the order Pleosporales, which predominantly consists of fungal pathogens that possess a necrotrophic lifestyle. Many have been shown or hypothesized to use host‐specific effectors to facilitate disease development (Friesen *et al*., 2008a). *Parastagonospora nodorum* uses a series of proteinaceous necrotrophic effectors (NEs) to confer virulence on wheat that carries matching dominant sensitivity/susceptibility genes (Tan *et al*., 2010). Thus far, three proteinaceous NEs have been identified at the gene level. *SnToxA* encodes a 13.2‐kDa mature protein which causes necrosis on wheat varieties that carry *Tsn1* located on wheat chromosome 5BL (Faris *et al*., 2010; Friesen *et al*., 2006). *SnTox1* encodes a 10.3‐kDa mature cysteine‐rich protein with a chitin‐like binding motif at the C‐terminus. Sensitivity to SnTox1 is conferred by the *Snn1* gene located on wheat chromosome 1BS (Liu *et al*., 2012, 2004). *SnTox3* encodes a cysteine‐rich, 17.5‐kDa mature protein. Sensitivity to SnTox3 is conferred by *Snn3‐B1* and *Snn3‐D1* located on wheat chromosomes 5BS and 5DS, respectively (Liu *et al*., 2009; Zhang *et al*., 2011). The role of NEs in *P. nodorum* is well defined. *Parastagonospora nodorum* mutants carrying effector gene deletions are poorly pathogenic on wheat cultivars with matching receptors (Friesen *et al*., 2006; Liu *et al*., 2009, 2012). Furthermore, quantitative trait locus (QTL) analyses have revealed that the *P. nodorum*–wheat pathosystem is riddled with further effector–host sensitivity gene interactions, such as SnTox2*–Snn2*, SnTox4*–Snn4*, SnTox5*–Snn5*, SnTox6–*Snn6* and SnTox7–*Snn7* (Friesen *et al*., 2008a; Gao *et al*., 2015; Shi *et al*., 2015; Tan *et al*., 2015). However, the genes that code for these NEs and the host dominant susceptibility receptors remain unidentified.

The acquisition of NE genes confers virulence and broadens the host range of a plant pathogen (Mehrabi *et al*., 2011). *Pyrenophora tritici‐repentis* is the causal agent of tan spot (syn. yellow spot) of wheat. Like *P. nodorum*, *Py. tritici‐repentis* relies on several NEs to facilitate host infection. Among these is a near‐identical ToxA‐encoding gene, called *PtrToxA*, which may have been acquired from *P. nodorum* through a horizontal gene transfer event (Ciuffetti *et al*., 1997; Friesen *et al*., 2006). The acquisition of *PtrToxA* in *Py. tritici‐repentis* has led to the emergence of a devastating disease of wheat that is the dominant wheat pathogen in Australia and is important in many other parts of the world (Oliver *et al*., 2016). Similar to the *P. nodorum* SnToxA NE, PtrToxA causes necrosis on *Tsn1* wheat (Liu *et al*., 2006). The horizontally transferred region is at least 11 kb and includes DNA upstream of *PtrToxA* which consists of a constitutive promoter (Lorang *et al*., 2001). It therefore seems likely that *SnToxA* and *PtrToxA* share a common signalling pathway. Evidence for an effector gene regulatory mechanism in *P. nodorum* was first reported by IpCho *et al*. (2010). Deletion of the APSES class transcription factor gene *SnStuA* in *P. nodorum* impaired vegetative development, abolished the ability to sporulate and caused a reduction in pathogenicity on wheat. Interestingly, the expression of *SnTox3* was down‐regulated in the *snstuA* mutants (IpCho *et al*., 2010).

The SNB pathosystem is dictated by effector–host sensitivity receptor epistatic interactions. Several studies have demonstrated that SnToxA–*Tsn1* (Friesen *et al*., 2008b), SnTox2–*Snn2* (Friesen *et al*., 2008b), SnTox5–*Snn5* (Friesen *et al*., 2012) and SnTox6–*Snn6* (Gao *et al*., 2015) interactions are epistatic to SnTox3–*Snn3*. However, the mechanism of epistasis is unclear. We have demonstrated recently that the SnTox1–*Snn1* interaction is epistatically dominant to SnTox3–*Snn3* in the establishment of SNB in popular Australian wheat varieties (Phan *et al*., 2016). When *SnTox1* was removed from *P. nodorum* via gene deletion, the SnTox3–*Snn3* interaction played an active role in SNB. Gene expression analysis indicated that *SnTox3* expression increased in the absence of *SnTox1*. The mechanism associated with the repression of *SnTox3* expression by *SnTox1* is unknown at this stage. Similarly in *Py. tritici‐repentis*, PtrToxA‐induced symptoms on wheat are epistatic to other effector‐induced symptoms (Manning and Ciuffetti, 2015). However, the mechanism underlying the epistatic regulation is unknown. The expression of NE genes in both fungi is complex and a knowledge of how these NE genes are regulated is still lacking.

The Pleosporales fungus *Alternaria brassicicola* is the causal agent of dark leaf spot on many *Brassica* species. A putative GAL4‐like transcription factor gene, *AbPf2*, of *A. brassicicola* was identified and characterized for its role in vegetative fitness and plant virulence. Mutants deleted in *AbPf2* were able to grow slowly on various Brassicae hosts, but were unable to cause any disease symptoms (Cho *et al*., 2013). RNA sequencing (RNAseq) analysis indicated that AbPf2 functions as a positive regulator of genes encoding secreted proteins, some of which possess hallmarks of effectors. However, evidence on the use of host‐specific effectors to mediate virulence on Brassicae remains unsubstantiated at this stage (Cho, 2015). In this study, we sought to determine the role of the Pf2 family in two economically important Pleosporales fungi in which proteinaceous NE–host susceptibility/sensitivity gene interactions are well defined (Ciuffetti *et al*., 2010; Oliver *et al*., 2012). A simple homology search in the *P. nodorum* genome identified a gene that is orthologous to *AbPf2*, which we refer to as *PnPf2*. A functional orthologue of *PnPf2* was also identified in the genome of *Py. tritici‐repentis*: *PtrPf2*. Functional analyses of *PnPf2* and *PtrPf2* provided a compelling insight into their role in the regulation of major effector genes and in conferring host‐specific virulence on wheat.

## Results

### Identification of the *AbPf2* orthologue in *P. nodorum* and *Py. tritici‐repentis*

**Figure 1:**
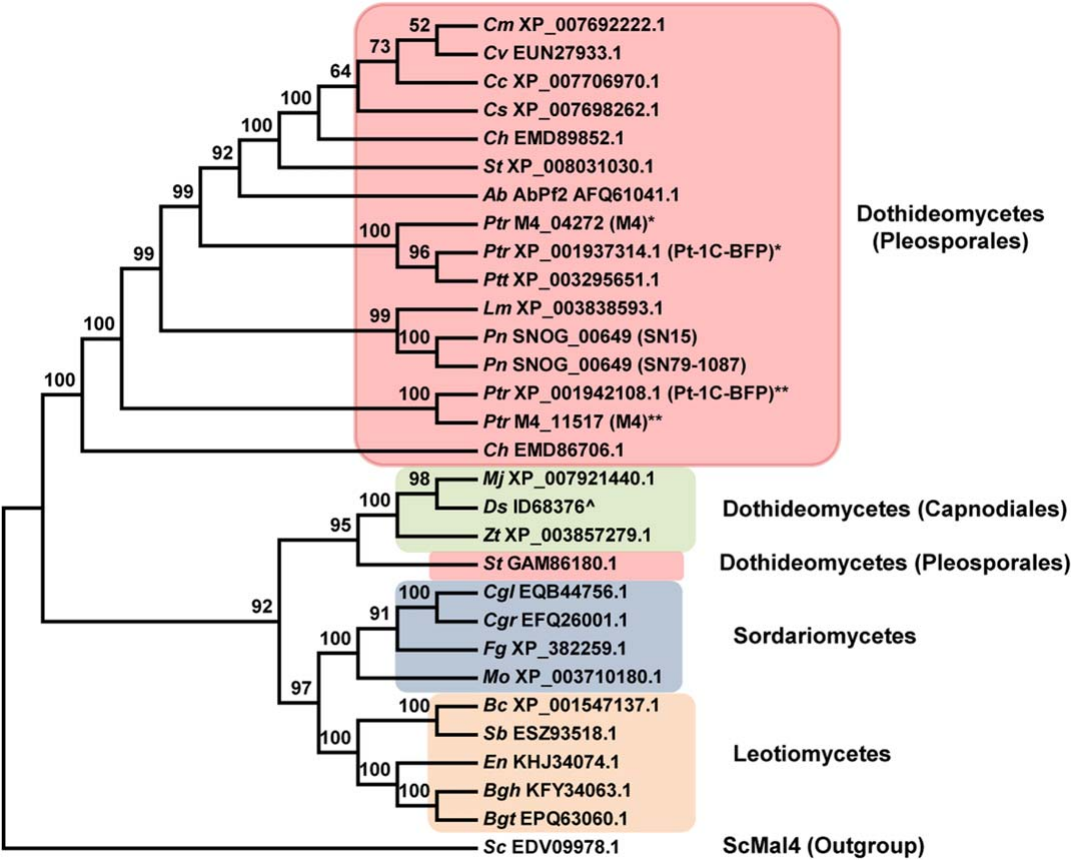
A bootstrap consensus phylogenetic tree showing the amino acid relationships between Pf2 orthologues and other putative transcription factors from various plant‐pathogenic fungi. ScMal4 from *Saccharomyces cerevisiae* was used as an outgroup. Percentage bootstrap values from 1000 repetitions are shown above the branches. Italicized prefix abbreviation of species: *Ab*, *Alternaria brassicicola*; *Bc*, *Botrytis cinerea*; *Bgh*, *Blumeria graminis* f. sp. *hordei*; *Bgt*, *Blumeria graminis* f. sp. *tritici*; *Cc*, *Cochliobolus carbonum*; *Cgl*, *Colletotrichum gloeosporioides*; *Cgr*, *Colletotrichum graminicola*; *Ch*, *Cochliobolus heterostrophus*; *Cm*, *Cochlioblus miyabeanus*; *Cs*, *Cochliobolus sativus*; *Cv*, *Cochliobolus victoriae*; *Ds*, *Dothiostroma septosporum*; *En*, *Erysiphe necator*; *Fg*, *Fusarium graminearum*; *Mf*, *Mycosphaerella fijiensis*; *Mo*, *Magnaporthe oryzae*; *Pn*, *Parastagonospora nodorum*; *Ptr*, *Pyrenophora tritici‐repentis*; *Ptt*, *Pyrenophora teres‐teres*; *Sb*, *Sclerotinia borealis*; *Sc*, *Saccharomyces cerevisiae*; *St*, *Setosphaeria turcica*; *Zt*, *Zymoseptoria tritici*. Amino acid sequences are deposited as Data S1. Where applicable, strains are indicated in parentheses. * and ** denote differences in annotation between M4 and Pt‐1C_BFP orthologues. ^^^Manually‐curated open reading frame (ORF) (Joint Genome Institute database).

The putative GAL4‐like transcription factor gene *AbPf2* of *A. brassicicola* functions as a virulence factor and positively regulates genes encoding effector‐like proteins (Cho *et al*., 2013). To determine whether *P. nodorum* possess an orthologue, the AbPf2 polypeptide sequence of 651 amino acids (Genbank Accession AFQ61041) was used to search the ‘Phaeosphaeria nodorum SN15’ Genbank non‐redundant (nr) protein database, and identified SNOG_00649 (Genbank Accession XP_001791330) as the best Blast hit. The corrected open reading frame (ORF) and amino acid sequence of SNOG_00649 are located at https://github.com/robsyme/Parastagonospora_nodorum_SN15 (Syme *et al*., 2016). The SNOG_00649 ORF consists of four RNAseq‐validated exons that encode a 652‐amino‐acid polypeptide (Syme *et al*., 2016). CD‐Blast analysis identified two conserved domains: a GAL4‐like Zn_2_Cys_6_ binuclear cluster domain from amino acid 11 to 46, and a fungal transcription factor regulatory middle homology region from amino acid 104 to 340 (Fig. S1, see Supporting Information). The SNOG_00649 polypeptide shares 76% amino acid identity with AbPf2. Phylogenetic analysis of the SNOG_00649 polypeptide indicated that it groups with AbPf2 and shares closest sequence resemblance to an orthologue from the canola blackleg fungus *Leptosphaeria maculans* (Fig. 1). Hereafter, we designate the SNOG_00649 gene as *PnPf2*. Interestingly, *Pf2* orthologues are exclusively derived from Pleosporales fungal pathogens.

In *Py. tritici‐repentis* wild‐type isolate M4 (race 1 strain from Australia; ToxA+, ToxB–, ToxC+), BlastP analysis identified two potential Pf2 orthologues: M4_04272 and M4_11517. M4_04272 was supported by RNAseq datasets derived from *in vitro*‐grown and infected wheat. M4_11517 was identified via *in silico* gene prediction as RNAseq data were unavailable (See et al., unpublished data). M4_04272 consists of four exons that encode a 650‐amino‐acid polypeptide. Nucleotide sequence analysis indicates that M4_04272 is near‐identical to its orthologue PTRG_06982 (Genbank ID: XP_001937314) from *Py. tritici‐repentis* Pt‐1C‐BFP, an isolate from the USA with a reported genome sequence (Manning *et al*., 2013). The former possess two missing nucleotides in the second intron. In addition, M4_04272 and PTRG_06982 possess different annotations at the 5′ region (Fig. S2A, see Supporting Information). M4_11517 consists of three exons that encode a 272‐amino‐acid polypeptide. The nucleotide gene sequence of M4_11517 shows 100% sequence identity to the gene sequence of PTRG_11777 (XP_001942108) of *Py. tritici‐repentis* Pt‐1C‐BFP, with the exception of one single nucleotide polymorphism (SNP) located outside the coding regions (Fig. S2B). The annotation of both ORFs differs at the 5′ region (Fig. S2B). CD‐Blast analysis (Marchler‐Bauer *et al*., 2015) of M4_04272 identified two conserved domains that are similar to predictions for AbPf2 and PnPf2 (Fig. S1). The fungal transcription factor regulatory middle homology region was predicted for the M4_11517 polypeptide and its orthologue PTRG_11777. The GAL4 domain was not predicted for either polypeptide using current gene annotations. PnPf2 and M4_04272 shared 76% amino acid identity, whereas similarity between PnPf2 and M4_11517 was much lower at 55% between the aligned amino acid residues. Thus, M4_04272 is the closest orthologue to *PnPf2* based on sequence analyses. Hereafter, M4_04272 is referred to as *PtrPf2*.

### *PnPf2* and *PtrPf2* are expressed during wheat infection

**Figure 2:**
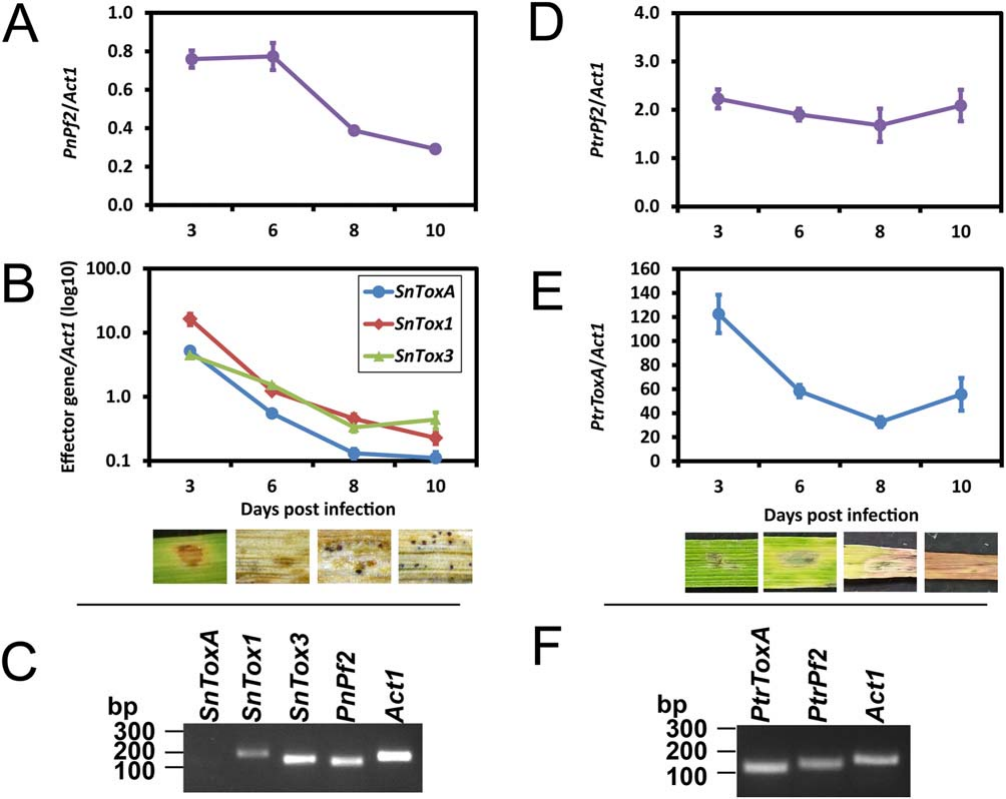
*Pf2* and effector genes are maximally expressed during early infection. *PnPf2* (A) and *SnToxA*, *SnTox1* and *SnTox3* (B) in *Parastagonospora nodorum* gene expression profile *in planta* (*n* = 3). *Parastagonospora nodorum*‐infected wheat tissues are shown at the time of sampling. (C) Real‐time polymerase chain reaction (PCR) analysis of *SnToxA*, *SnTox1*, *SnTox3*, *PnPf2* and *Act1* expression in SN15 during growth in Fries 3 broth. *In planta* gene expression profile of *PtrPf2* (D) and *PtrToxA* (E) (*n* = 3). *Pyrenophora tritici*‐*repentis*‐infected wheat tissues are shown at the time of sampling. Error bars are shown as the standard error of the mean in all graphs. (F) Real‐time PCR analysis of *PtrToxA*, *PtrPf2* and *PtrAct1* expression in *P. tritici‐repentis* during growth in Fries 3 broth. The experiment was performed using pooled biological triplicate samples.

The expression profile of *PnPf2* during wheat infection was determined using quantitative real‐time polymerase chain reaction (PCR). Infection time points, presented as days post‐infection (dpi), were chosen to represent all major stages of infection, as described by Solomon *et al*. (2006c): early penetration (3 dpi), colonization (6 dpi) and pycnidiation (8 and 10 dpi). The expression level of *PnPf2* was maximal at 3 and 6 dpi, but decreased during the onset of pycnidiation (Fig. 2A). The expression of *SnToxA*, *SnTox1* and *SnTox3* was also examined. All three effector genes demonstrated maximal expression at 3 dpi, followed by a dramatic decrease in expression from 6 dpi which coincided with tissue necrosis (Fig. 2B). *SnTox1*, *SnTox3* and *PnPf2*, but not *SnToxA*, transcripts were also readily detected during growth in Fries 3 broth (Fig. 2C).

For *Py. tritici‐repentis*, *PtrPf2* was constitutively expressed between 3 and 10 dpi on wheat (Fig. 2D). Similar to *SnToxA*, the expression of *PtrToxA* was maximal during early infection (3 dpi), but gradually decreased from 6 dpi onwards, which coincided with the onset of chlorosis and necrosis of the host tissue (Fig. 2E). *PtrPf2* and *PtrToxA* were expressed during axenic growth in Fries 3 broth (Fig. 2F).

### Comparative analysis of the *ToxA* promoter region in *P. nodorum* and *Py. tritici‐repentis* reveals a high level of genetic polymorphism

**Figure 3:**
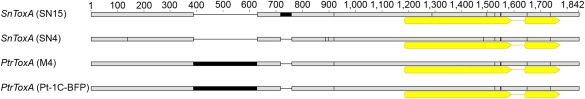
Nucleotide alignment of the putative promoter region of *ToxA* (yellow) from *Parastagonospora nodorum* (SN15 and SN4) and *Pyrenophora tritici‐repentis* (M4 and Pt‐1C‐BFP) showing extensive nucleotide polymorphism at the putative 5′ promoter region. Nucleotide sequences are described in Fig. S3 (see Supporting Information).

As *SnToxA* and *PtrToxA* demonstrated differential expression during *in vitro* growth, we hypothesized that genetic polymorphisms were present within the promoter region of the *Pf2* orthologues. We compared the 5′ untranslated region (UTR) of two *P. nodorum* isolates (SN15, Australia and SN4, USA) with that of two *Py. tritici‐repentis* isolates (M4, Australia and Pt‐1C‐BFP, USA). Sequence alignment of the 5′ ToxA UTR promoter region revealed evidence of genetic polymorphism, including sectional deletions and SNPs between the two species and *P. nodorum* strains (Fig. 3). This may account for the differences in *SnToxA* and *PtrToxA in vitro* expression profiles.

### *PnPf2* is deleted in *P. nodorum* SN15

As *PnPf2* is expressed during infection, we deleted the gene in the *P. nodorum* SN15 wild‐type background using targeted gene deletion to determine its function in virulence on wheat (Fig. S4, see Supporting Information). Mutants that carry the appropriate gene deletion were identified using PCR. A robust quantitative PCR method was used to determine the copy number of *PnPf2* deletion constructs in all transformants to identify appropriate mutants that carry single‐copy integration (Solomon *et al*., 2008). Consequently, strains deleted in *PnPf2* (*pf2‐63* and *pf2‐69*) and an ectopic (Ect) mutant were retained for phenotypic characterization (Table 1). The Ect strain contains a selectable marker insertion elsewhere in the genome other than in *PnPf2*, and thus all assayed phenotypes should be similar to SN15.

**Table 1:** Fungal strains used throughout this study.

Strain	Description	Source
SN15	*Parastagonospora nodorum* wild‐type	Department of Agriculture, Western Australia
Ect	*Parastagonospora nodorum* SN15 ectopic transformant	This study
*pf2‐63*	SN15 deleted in *PnPf2*	This study
*pf2‐69*	SN15 deleted in *PnPf2*	This study
*pf2*::*PnPf2*	*pf2‐69* complemented with *PnPf2*	This study
*pf2‐tox1‐2*	*pf2‐69* deleted in *SnTox1*	This study
*pf2‐tox1‐6*	*pf2‐69* deleted in *SnTox1*	This study
*pf2‐tox1‐16*	*pf2‐69* deleted in *SnTox1*	This study
*pf2‐Hph‐5*	*pf2‐69* carrying an ectopic *Hph* integration	This study
*tox1‐6*	SN15 deleted in *SnTox1*	Phan *et al*. (2016)
M4	*Pyrenophora tritici‐repentis* wild‐type	Meckering, Western Australia
E‐1	*Pyrenophora tritici‐repentis* M4 ectopic transformant	This study
*pf2‐a*	M4 deleted in *PtrPf2*	This study
*pf2‐b*	M4 deleted in *PtrPf2*	This study
*pf2‐c*	M4 deleted in *PtrPf2*	This study

A preliminary analysis of *pf2‐63* and *pf2‐69* indicated that vegetative growth on an undefined solid medium was indistinguishable from that of SN15 and Ect (Fig. S5A, see Supporting Information). Furthermore, no significant differences in asexual sporulation were observed in the absence of *PnPf2* (Fig. S5B). This suggests that *PnPf2* plays a dispensable role in vegetative growth.

### *PnPf2* deletion reduces *P. nodorum* virulence on *Tsn1* and *Snn3* wheat cultivars

**Figure 4:**
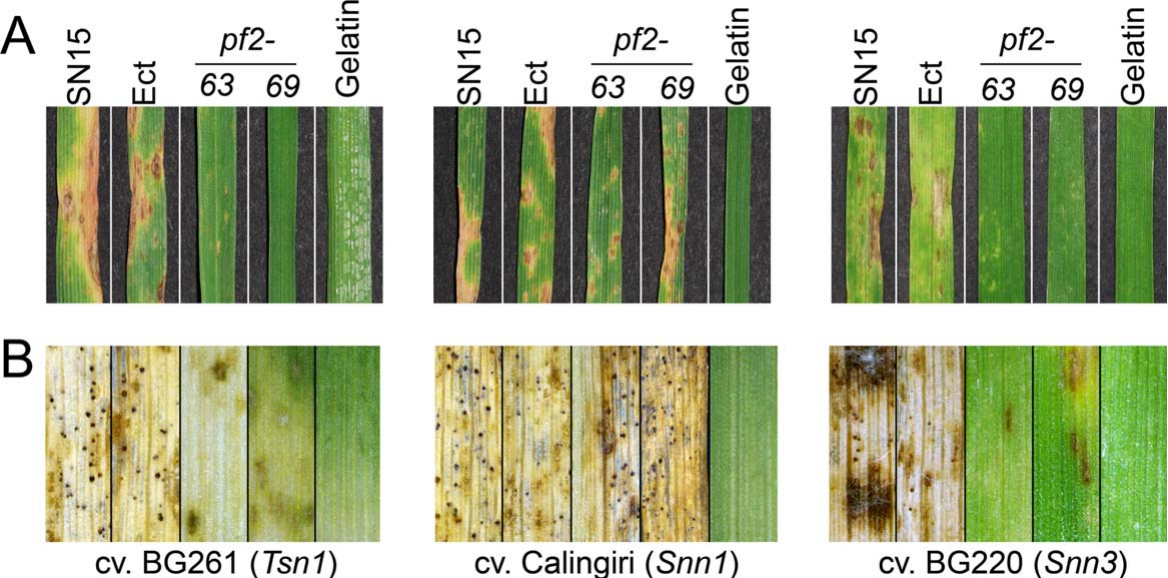
*PnPf2* is associated with host‐specific virulence on wheat. A whole‐plant infection assay on wheat cultivars possessing *Tsn1*, *Snn1* or *Snn3* genotypes. (A) Development of necrosis after 7 days post‐infection (dpi) and (B) pycnidia after 14 dpi. Genetic complementation restored virulence on BG261 and BG220 comparable with that on SN15 (Fig. S6).

The role of *PnPf2* in virulence was examined using a whole‐plant spray infection assay. Wheat cultivars BG261 (*Tsn1*, *snn1*, *snn3*), Calingiri (*tsn1*, *Snn1*, *snn3*) and BG220 (*tsn1*, *snn1, Snn3*) were used as hosts as they contain differential sensitivities to all three effectors (Liu *et al*., 2009, 2012; Tan *et al*., 2014). *Parastagonospora nodorum pf2‐63* and *pf2‐69* were able to cause full infection on Calingiri, but gave no significant necrosis on BG261 and BG220 (Fig. 4A). We tested the disease susceptibility of two other independent *Snn1‐* and *Snn3*‐specific wheat cultivars: Chinese Spring (*Snn1*, *tsn1*, *snn3*) and Wyalkatchem (*tsn1*, *snn1*, *Snn3*) (Tan *et al*., 2014). *PnPf2* deletion impaired virulence on Wyalkatchem, but remained fully pathogenic on Chinese Spring (Fig. S6, see Supporting Information). Apart from BG261, we were unable to source another independent *Tsn1* wheat cultivar that was insensitive to SnTox1 and SnTox3.

*Parastagonospora nodorum* produces asexual pycnidiospores in pycnidia during late infection to facilitate secondary local infection. *Parastagonospora nodorum* SN15 and Ect were able to produce pycnidia on BG261, Calingiri and BG220. Not surprisingly, *pf2‐63* and *pf2‐69* were only able to form pycnidia on Calingiri (Fig. 4B). We then genetically complemented *pf2‐69* with *PnPf2* and its native promoter and terminator to give *P. nodorum pf2*::*PnPf2*. Genetic complementation restored full virulence on *Tsn1* and *Snn3* wheat cultivars (Fig. S7, see Supporting Information). This suggests that *PnPf2* deletion abolished SnToxA and SnTox3 production, and thus the fungus cannot complete its infection life cycle on hosts that carry *Tsn1* or *Snn3* only.

### *PnPf2* regulates *SnToxA* and *SnTox3* expression

**Figure 5:**
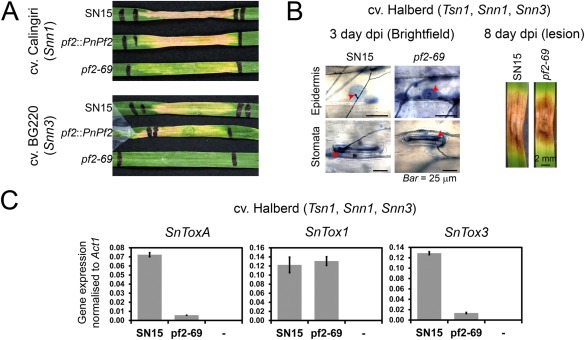
*PnPf2* is a positive regulator of *SnToxA* and *SnTox3* expression. (A) Chlorotic necrotic symptoms of wheat cv. Calingiri (*Snn1*) and BG220 (*Snn3*) infiltrated with culture filtrates of SN15 and *pf2‐69*. Infiltration of BG261 (*Tsn1*) with culture filtrates of all strains did not result in chlorosis (data not shown). (B) Wheat cv. Halberd is susceptible to SN15 and *pf2‐69*. At 3 days post‐infection (dpi), trypan blue staining revealed that *pf2‐69* attempts to enter the host through the stomata and epidermis via hyphopodia, similar to SN15 (red arrows). At 8 dpi, the necrotic lesion caused by *pf2‐69* is comparable with that of SN15. (C) Halberd was used as a host to determine the expression of *SnToxA*, *SnTox1* and *SnTox3* in SN15 and *pf2‐69* at 3 dpi. –, Tween non‐infection control. Error bars are shown as the standard error of the mean. Quantitative real‐time polymerase chain reaction was performed with biological triplicates.

To test the hypothesis that *PnPf2* functions as a positive regulator of *SnToxA* and *SnTox3* expression, we performed a series of effector infiltration experiments using culture filtrates derived from wild‐type and gene deletion strains. SnTox1 activity from the culture filtrate of *pf2‐69* was detected on Calingiri (*Snn1*). However, infiltration of BG220 (*Snn3*) with the culture filtrate derived from *pf2‐69* did not result in necrosis (Fig. 5A). SnTox3 activity was restored in the genetically complemented strain *pf2*::*PnPf2*.

SnToxA activity cannot be assayed in culture filtrates as *P. nodorum* SN15 does not express *SnToxA in vitro* (Tan *et al*., 2015). To determine whether PnPf2 regulates the expression of *SnToxA*, we used wheat cv. Halberd which is sensitive to SnToxA, SnTox1 and SnTox3 (Tan *et al*., 2015), and demonstrated strong susceptibility to *pf2‐69* (Fig. 5B). At 3 dpi, *SnToxA* and *SnTox3* expression was strongly reduced compared with that in SN15, whereas *SnTox1* expression was unaffected (Fig. 5C). This suggests that *PnPf2* functions as a regulator of *SnToxA* and *SnTox3*, but not *SnTox1*, expression.

### Biochemical complementation with SnToxA and SnTox3 restores virulence in a *PnPf2* deletion background

**Figure 6:**
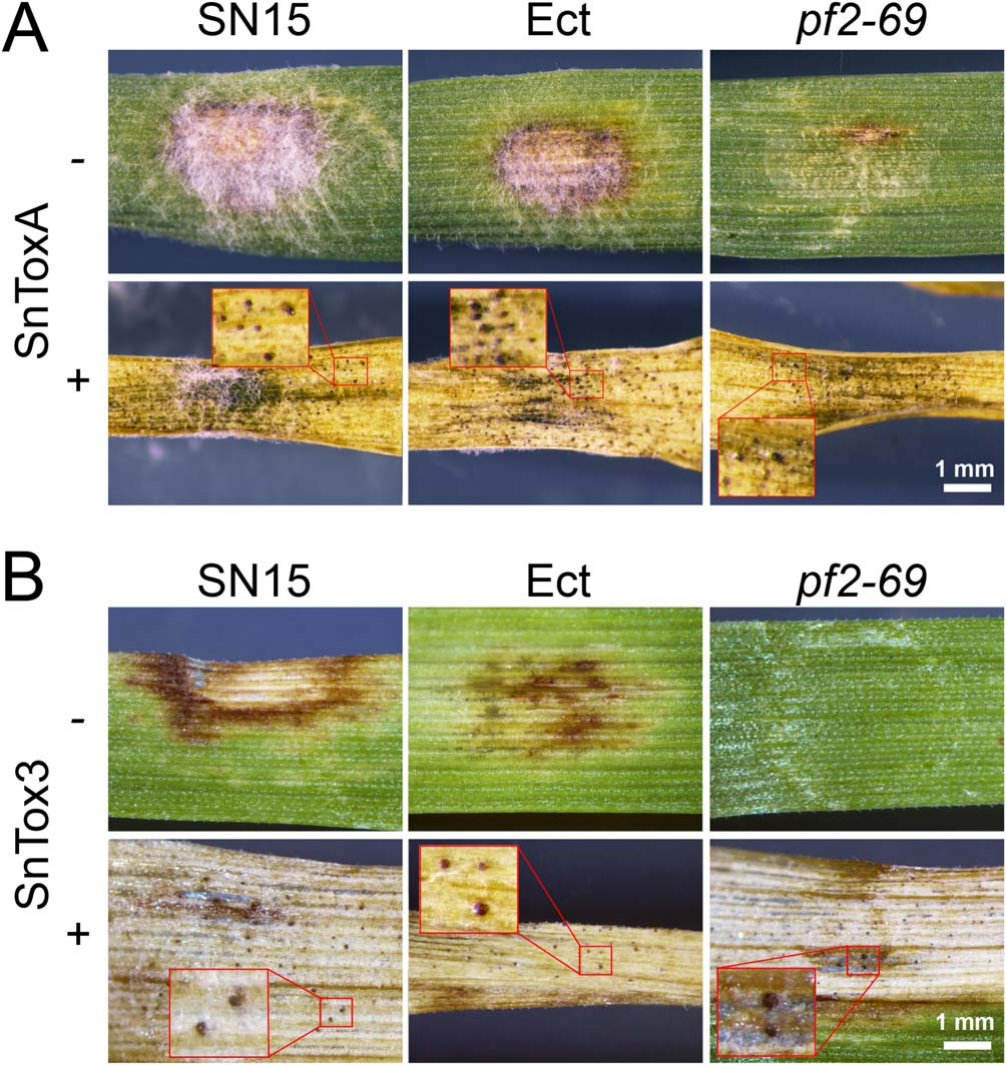
SnToxA and SnTox3 pre‐infiltration restores virulence. Detached leaf assay of BG261 (*Tsn1*) (A) and BG220 (*Snn3*) (B) infected with SN15, Ect and *pf2‐69* with and without effector pre‐infiltration. Pycnidiation is an indication of virulence in the effector pre‐infiltrated treatments (+).

To test whether the reduction in virulence caused by the *PnPf2* deletion is or is not a pleiotropic effect, we infected BG261 and BG220 which had been pre‐infiltrated with SnToxA and SnTox3, respectively, 24 h previously. Pycnidiation was used as a measure of virulence. Pre‐infiltration of SnToxA on BG261 restored *pf2‐69* virulence on *Tsn1* wheat (Fig. 6A). Similarly, pre‐infiltration of SnTox3 on BG220 restored the ability of *pf2‐69* to proliferate and produce pycnidia (Fig. 6B). This further indicates that the reduction in virulence of *pf2* deletion mutants on BG261 and BG220 can be attributed solely to the inability to produce sufficient levels of SnToxA and SnTox3.

### Deletion of *SnTox1* in the *pnpf2* background eliminates virulence on *Snn1* wheat

**Figure 7:**
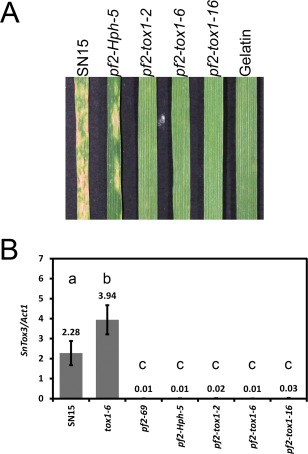
*SnTox1* deletion in a *pnpf2* background abolishes virulence on *Snn1* wheat. (A) *SnTox1* and *PnPf2* are required for virulence on Calingiri (*tsn1*, *Snn1*, *snn3*). (B) Quantitative real‐time polymerase chain reaction analysis of *SnTox3* expression during *in vitro* growth. *SnTox1* deletion does not restore *SnTox3* expression in the *pf2* background. Standard error bars are shown. Normalized *SnTox3/Act1* values are provided above the error bars. Levels not connected by the same letter are significantly different (*P* < 0.05) based on analysis of variance. The experiment was performed with biological replicates (*n* = 6).

It was anticipated that *SnTox1* deletion in the *pf2* background would result in a reduction in the virulence of *P. nodorum* on *Snn1* wheat. *Parastagonospora nodorum* strains deleted in *SnTox1* were created using the *pf2‐69* background. PCR analysis identified three appropriate *SnTox1* knockout strains (*pf2‐tox1‐2*, *pf2‐tox1‐6* and *pf2‐tox1‐16*) and one ectopic mutant harbouring the *Hph* selectable marker outside of *SnTox1* (Fig. S8, see Supporting Information). As expected, all *pf2‐tox1* strains were non‐pathogenic on the *Snn1* wheat cultivar Calingiri (Fig. 7A).

### PnPf2 is dominant over SnTox1 epistatic regulation of *SnTox3* expression

We have demonstrated previously that the SnTox1–*Snn1* interaction is epistatic to SnTox3*–Snn3*. Hence, no effect of SnTox3–*Snn3* is observed during SNB unless infection is carried out with a *P. nodorum* strain deleted in *SnTox1* (*tox1‐6*) (Phan *et al*., 2016). A significant increase in *SnTox3* expression was observed in *P. nodorum tox1‐6* during wheat infection and growth *in vitro*. Thus, *SnTox1* functions as a negative regulator of *SnTox3* expression, unlike PnPf2. We then sought to determine whether PnPf2 or SnTox1 function as the dominant regulator of *SnTox3* expression using quantitative real‐time PCR analysis of *in vitro*‐grown *P. nodorum* mutants that lacked *PnPf2* and/or *SnTox1*. *SnTox3* expression was observed in SN15 and *tox1‐6*. However, *SnTox3* transcripts were barely detectable in all *P. nodorum* strains that lacked *PnPf2*, including those that carried the *SnTox1* double deletion (Fig. 7B). This suggests that PnPf2 plays a dominant role over SnTox1 in the regulation of *SnTox3* expression. We used QTL mapping of wheat to determine whether the SnTox3–*Snn3* interaction is observed during SNB in the absence of *SnTox1*. *Parastagonospora nodorum pf2‐tox1‐6* was used to infect seedlings of a double haploid (DH) mapping population constructed from a cross between Calingiri (*tsn1*, *Snn1*, *snn3*) and Wyalkatchem (*tsn1*, *snn1*, *Snn3*). The mutant was generally much less pathogenic on the DH population than on SN15, but comparable with *tox1‐6* (Fig. S9, see Supporting Information). As expected, SnTox3‐responsive QTLs on chromosomes 5BS (*Snn3*) and 4BL were not detected from *pf2‐tox1‐6* infection (Table 2). Previously detected SNB QTLs on 2DS and 3AL were observed from *pf2‐tox1‐6* infection (Table 2). In addition, new SNB QTLs on 2AS and 6A were detected.

**Table 2:** *PnPf2* deletion abolishes the SnTox3–*Snn3* interaction during Septoria nodorum blotch (SNB).

Chromosome arm	QTL	Locus/QTL flanking markers	LOD	*R* ^2^	Effect
2AS2	Qsnb.cur‐2AS2	wmc382a ‐ barc124a	5.7	15	0.90
2DS	Qsnb.cur‐2DS	cfd36 ‐ wPt‐669517	6.4	17	−0.94
3AL	Qsnb.cur‐3AL	tPt‐1143 ‐ wPT‐4859	6.0	16	0.90
6A1	Qsnb.cur‐6AS	gpw4329 ‐ wPt‐4270	6.0	16	0.90

A summary of quantitative trait loci (QTLs) identified in this study from *Parastagonospora nodorum pf2‐tox1‐6* seedling infection. Flanking markers, logarithm of odds (LOD) scores, phenotype contribution (*R* ^2^) and parental effect of these QTLs are shown. Positive and negative effects indicate that the allele was inherited from Calingiri and Wyalkatchem, respectively. A genetic map is provided in Fig. S10 (see Supporting Information).

### *PtrPf2* regulates *PtrToxA* expression and virulence

**Figure 8:**
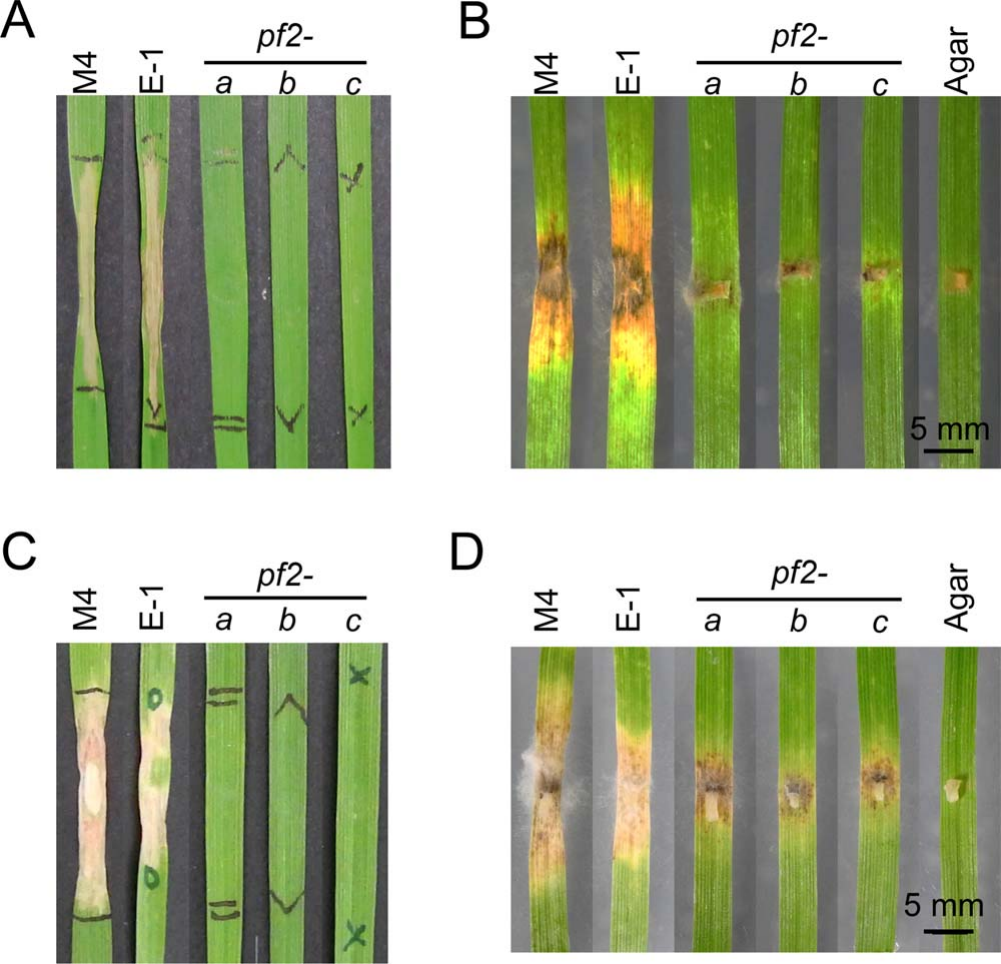
PtrPf2 regulates the production of PtrToxA and an undiscovered necrosis‐inducing factor(s). Culture filtrate activity (A) and virulence (B) of *Pyrenophora tritici‐repentis* M4 wild‐type, E‐1 and *PtrPf2* deletion mutants on wheat cv. BG261 (*Tsn1*). Culture filtrate activity (C) and virulence (D) of *P. tritici‐repentis* M4 wild‐type, E‐1 and *PtrPf2* deletion mutants on wheat cv. Machete (*tsn1*).

As *PtrToxA* may have been acquired through a horizontal gene transfer event from *P. nodorum*, we hypothesized that *PtrPf2* functions similarly to regulate its expression in *Py. tritici‐repentis*. To test this, we deleted *PtrPf2* from *Py. tritici‐repentis* M4 using homologous recombination (Moffat *et al*., 2014) and determined the effect on *PtrToxA* expression and virulence. An ectopic (E‐1) and three *PtrPf2* deletion strains (*pf2‐a*, *pf2‐b* and *pf2‐c*) were selected for phenotypic analyses (Fig. S11, see Supporting Information; Table 1). As PtrToxA is secreted into the culture filtrate of *Py. tritici‐repentis* (unlike *P. nodorum* SnToxA) (Moffat *et al*., 2014; Tan *et al*., 2014), we could determine whether PtrPf2 regulates PtrToxA production by infiltration experiments into wheat cv. BG261 (*Tsn1*). Culture filtrates from M4 and E‐1 produced a strong necrotic response after 4 days, but no visible signs of chlorosis or necrosis were seen with *pf2‐a*, *pf2‐b* and *pf2‐c* (Fig. 8A). The role of *PtrPf2* in virulence was then examined using a detached leaf assay on BG261. As conidiation was abolished in *pf2‐a*, *pf2‐b* and *pf2‐c*, the infection assay was inoculated with mycelial plugs and lesions were allowed to develop. As expected, *Py. tritici‐repentis pf2‐a*, *pf2‐b* and *pf2‐c* displayed a near‐complete loss of virulence on BG261 compared with M4 and E‐1 (Fig. 8B). This strongly indicates that *PtrPf2* positively regulates *PtrToxA* in *Py. tritici‐repentis*. The near‐complete loss in virulence in *Tsn1* wheat was independently confirmed using another wheat cultivar, Yitpi, which is insensitive to ToxA (Fig. S12, see Supporting Information). Quantitative real‐time PCR analysis indicated that the expression of *PtrToxA* is barely detectable in the *ptrpf2* mutants, thus indicating that the loss of detectable PtrToxA activity is associated with transcriptional down‐regulation (Table 3). *Pyrenophora tritici‐repentis* M4 is a race 1 pathogen and thus produces the ToxC effector that induces chlorosis (Moffat *et al*., 2014). The wheat cv. 6B365 is sensitive to the chlorosis‐inducing effect of ToxC, but is insensitive to ToxA (Moffat *et al*., 2014). We infiltrated culture filtrates of M4, E1 and all *ptrpf2* deletion strains into wheat cv. 6B365 with inconsistent results (data not shown). However, the Australian commercial wheat cv. Machete (insensitive to ToxA) gave distinct chlorosis with M4 and E‐1, but not with the *ptrpf2* deletion strains (Fig. 8C). At this stage, the identity of the strong chlorosis‐inducing factor is unknown. Virulence assay indicated that *Py. tritici‐repentis* mutants deleted in *PtrPf2* cause small lesions on wheat cv. Machete which resemble a hypersensitive response (Fig. 8D).

**Table 3:** PtrPf2 is a positive regulator of *PtrToxA* expression.

Strain	*PtrToxA/PtrAct1*	Standard error of the mean
M4	29474.32	28241.12
E‐1	9640.70	4070.35
*pf2‐a*	0.82	0.03
*pf2‐b*	2.13	1.19
*pf2‐c*	3.19	1.48

Quantitative real‐time polymerase chain reaction analysis of *PtrToxA* expression (normalized to *PtrAct1* expression) in *Pyrenophora tritici‐repentis* M4, E‐1 and *ptrpf2* mutants grown *in vitro*. The experiment was performed with biological replicates.

### *PtrPf2* is required for conidiation and normal vegetative growth

Vegetative growth of *ptrpf2* strains on solid V8‐PDA medium was compared with that of M4 and E‐1. All *ptrpf2* strains produced fewer aerial hyphae and the colony morphology lacked radial crevassing, relative to M4 and E‐1 (Fig. S13, see Supporting Information). Conidiation was abolished in all *ptrpf2* stains (Table S1, see Supporting Information). Thus, *PtrPf2* plays a substantial regulatory role in vegetative morphogenesis, not seen by *PnPf2* in *P. nodorum*. As a result of the resulting phenotype in the *ptrpf2* mutants, genetic complementation cannot be performed using established methods as transformation requires protoplasts derived from germinated conidiospores (Ciuffetti *et al*., 1997; Moffat *et al*., 2014). We attempted to generate sufficient quantities of protoplasts using macerated fungal hyphae, but were unsuccessful.

## Discussion

We have dissected the role of a member of a unique class of GAL4‐like Zn_2_Cys_6_ transcription factors in two major fungal pathogens of wheat in the regulation of effector expression and host genotype‐specific virulence. Deletion of the *Pf2* orthologue in both *P. nodorum* and *Py. tritici‐repentis* abolished ToxA expression and virulence on *Tsn1* wheat. In addition, *PnPf2* deletion in *P. nodorum* caused a strong reduction in *SnTox3* expression and subsequently reduced virulence on *Snn3* wheat. To our knowledge, this is the first study to demonstrate that a common regulatory mechanism exists between two closely related fungi that regulate functionally conserved effector(s). This enhances our knowledge of the two pathosystems and opens up a new route to discover further effectors in Pleosporales fungi. At this stage, it is not known whether PnPf2 and PtrPf2 function as direct or indirect regulators of effector gene expression in *P. nodorum* and *Py. tritici‐repentis*, respectively.

The regulation of effector gene expression in phytopathogenic fungi is a relatively unexplored area of phytopathology. Soyer *et al*. (2015) used RNA interference to silence *LmStuA*, which encodes a putative helix–loop–helix transcription factor belonging to the APSES protein family, in the canola blackleg pathogen *Leptosphaeria maculans*. Silencing of *LmStuA* expression abolished *L. maculans* virulence on canola. Transcriptome analysis identified the down‐regulation of expression of three avirulence genes: *AvrLm1*, *AvrLm4‐7* and *AvrLm6*. Perturbation of StuA‐mediated signalling also resulted in abnormal vegetative growth, loss of sporulation and virulence in *P. nodorum*. In addition, the expression of *SnTox3* was significantly reduced and could be attributed to pleiotropic effects caused by *StuA* inactivation (IpCho *et al*., 2010; Soyer *et al*., 2015). However, it is still not known whether *SnTox3* in *P. nodorum* and *AvrLm4‐7*, *AvrLm1* and *AvrLm6* in *L. maculans* are subjected to direct or indirect regulation by *SnStuA* and *LmStuA*, respectively.

A peculiar phenomenon associated with effector gene regulation in *P. nodorum* has been demonstrated recently by Phan *et al*. (2016). The SnTox1–*Snn1* interaction is epistatic over SnTox3–*Snn3* in the development of SNB. Removal of *SnTox1* in *P. nodorum* unmasked the SnTox3–*Snn3* interaction during SNB. The mechanism of the epistatic interaction can be explained, in part, by an increased expression of *SnTox3*. In this study, we independently validated that SnTox1 is a negative regulator of *SnTox3* expression, but is recessive to *PnPf2*. This is demonstrated by the lack of expression of *SnTox3* in the *pnpf2* background *in vitro* and the SnTox3–*Snn3* interaction being absent during SNB. The exact mechanism by which SnTox1 and PnPf2 function to co‐regulate *SnTox3* expression has yet to be elucidated.

*Wor1* (*W*hite‐*o*paque *r*egulator 1) encodes a nuclear protein that was first characterized as a master regulator of morphological switching and virulence in *Candida albicans*, an opportunistic fungal pathogen of humans (Huang *et al*., 2006). Recently, the role of *Wor1* orthologues has been examined in several plant‐pathogenic fungi in terms of the virulence and regulation of secreted proteins, some of which possess effector‐like characteristics (Michielse *et al*., 2009; Mirzadi Gohari *et al*., 2014; Okmen *et al*., 2014; Santhanam and Thomma, 2013). In the vascular wilt fungus *Fusarium oxysporum* f. sp. *lycopersici*, the *Wor1* orthologue *Sge1* is essential for virulence on tomatoes and functions as a positive regulator of four effector genes: *SIX1*, *SIX2*, *SIX3* and *SIX5* (Michielse *et al*., 2009). Blast analysis indicates that *P. nodorum* (SNOG_30643) and *Py. tritici‐repentis* (PTRG_01056) possess a *Wor1* orthologue. It remains to be determined whether the *Wor1* orthologue plays a concerted role with Pf2 in the regulation of effector gene expression in *P. nodorum* and *Py. tritici‐repentis*.

This study has demonstrated that, in both *P. nodorum* and *Py. tritici‐repentis*, the Pf2 transcription factor plays a crucial role in the regulation of *ToxA* expression and possibly other effectors. However, under *in vitro* conditions, *ToxA* is highly expressed in *Py. tritici‐repentis*, but is undetectable in *P. nodorum* (unattainable under our laboratory conditions) (Moffat *et al*., 2014; Tan *et al*., 2014). We have yet to identify the signal that induces *SnToxA* expression *in vitro*. In addition, evidence of extensive genetic polymorphism at the 5′ ToxA UTR between *P. nodorum* and *Py. tritici‐repentis* was observed (Fig. 3). At this stage, the link between *ToxA* expression and genetic polymorphism in the 5′ UTR between the two pathogens is unclear, but this observation has opened up opportunities to explore possible links.

*PtrPf2* plays a critical role in vegetative growth and conidiospore production in *Py. tritici‐repentis*, but not in *P. nodorum*. The *pnpf2* mutants retain the ability to infect wheat cultivars that possess *Snn1* (i.e. Halberd, Chinese Spring and Calingiri), but not cultivars that lack *Snn1*. Microscopy analysis revealed that *pf2‐69*, like SN15, was able to infect the *Snn1* host through direct entry via the stomata and epidermal penetration using hyphopodia (Solomon *et al*., 2004) (Fig. 5B). This strongly indicates that *PnPf2* does not play a significant role in the regulation of the basic machinery used by *P. nodorum* to facilitate infection on wheat, other than effector‐assisted entry.

The discovery of a functionally conserved transcription factor class in Pleosporales fungi with regard to its role in the regulation of small secreted protein and effector gene expression poses several key research questions. First, do *PnPf2* and *PtrPf2* regulate the expression of small secreted proteins and effectors other than *ToxA* and *SnTox3*? A preliminary study using a whole‐plant infection assay demonstrated that the virulence of *pf2‐63* and *pf2‐69* on wheat cv. BG223 (*Snn2*, *tsn1*, *snn1*, *snn3*) was greatly reduced (Fig. S14, see Supporting Information). *Snn2* is located on chromosome 2DS (Friesen *et al*., 2007). Hence, it is unlikely that the 2DS QTLs detected in this study are associated with SnTox2 sensitivity. In addition, the removal of SnTox1 in *pf2‐69* resulted in a complete loss of virulence on wheat cv. Calingiri, which has been previously demonstrated to show susceptibility to a mutant strain of *P. nodorum* SN15 carrying *SnToxA*, *SnTox1* and *SnTox3* deletions (Tan *et al*., 2015). Therefore, it is reasonable to conclude that PnPf2 regulates *SnTox2* and other novel effectors. We have recently commenced extensive functional characterization of *PnPf2* and *PtrPf2* using chromatin immunoprecipitation sequencing, comparative transcriptomic and proteomic approaches to identify targets such as potential new effectors. Second, do Pf2 orthologues in other Pleosporales regulate the expression of effector genes? Third, what is the function of the second *Pf2*‐like gene M4_11517 in *Py. tritici‐repentis*? Fourth, what is the evolutionary history of the Pf2 transcription factors in the Pleosporales? Finally, what are the signals that activate *Pf2* expression? The identification of these signals is paramount in the formulation of strategies to simultaneously shut down the expression of multiple effector genes from two major fungal pathogens of wheat.

## Experimental Procedures

### Phylogenetic tree construction

Phylogenetic analysis of PnPf2 (SNOG_00649) and PtrPf2 (M4_04272) with orthologues and homologues from other fungal pathogens was performed using MEGA6 software (Tamura *et al*., 2013). Orthologues and homologues were selected from fungal pathogens based on the top 100 BlastP hits to AbPf2 via the nr database. Manual annotations were conducted for incorrectly annotated genes, where stated. All polypeptide sequences were aligned using ClustalW set at the ‘Gonnet’ weight matrix, ‘gap opening penalty’ of 10, ‘gap extension penalty’ of 20 and ‘gap separation distance’ of 5. The phylogenetic tree was constructed using the ‘neighbour‐joining’ algorithm, ‘p‐distance’ substitution model. Bootstrap analysis set at 1000 repetitions was used to test the statistical significance of the phylogenetic tree.

### Fungal culture

All fungal strains were maintained on V8‐PDA agar [150 mL/L Campbell's V8 juice, 3 g/L CaCO_3_, 10 g/L Difco potato dextrose agar (PDA) and 10 g/L agar] at 21 °C under a 12‐h photoperiod. All fungal strains used in this study are described in Table 1.

### The production of NEs and plant infiltration

*Parastagonospora nodorum* and *Py. tritici‐repentis* culture filtrates containing NEs were produced from growth in Fries 3 medium broth (Liu *et al*., 2004). Culture filtrates containing effectors were sequentially filtered using gauze, miracloth, Whatman paper and 0.22‐μm sterilizers.

For heterologous effector production, *SnTox3* was expressed in *Pichia pastoris* using the pGAPzA expression vector (Liu *et al*., 2009). *SnToxA* was expressed in *Escherichia coli* BL21E using the pET21a expression vector (Tan *et al*., 2012). Protein preparations containing the expressed effector were harvested and desalted with 10 mm sodium phosphate buffer, pH 7.0, prior to infiltration into the first leaf of 2‐week‐old wheat seedlings using a needleless 1‐cm^3^ syringe. Infiltrated leaves were monitored for necrotic development over 7 days post‐infiltration.

### Gene expression analysis

For *P. nodorum*, RNA isolation and *in planta* gene expression analysis were performed as described previously with minor modifications (Solomon *et al*., 2003). Briefly, detached wheat leaves (cv. Halberd) maintained in 75 mg/L benzimidazole agar were inoculated with 1 × 10^6^ pycnidiospores in 0.02% Tween 20 to facilitate infection. Lesions were excised from infected wheat, freeze dried and subjected to RNA extraction using TRIzol reagent (Invitrogen, La Jolla, CA, USA), Dnase‐treated and reverse transcribed as described previously (Tan *et al*., 2008). Quantitative real‐time PCR was performed using a Quantitect SYBR Green RT‐PCR kit (Qiagen, Valencia, CA, USA) and a Bio‐Rad (Hercules, CA, USA) CFX96 system employing SN15 genomic DNA as a quantitative standard. The primer pair Pf2qPCRf and Pf2qPCRr was used to amplify a 130‐bp region of *PnPf2*. The primer pair ToxAqPCRf and ToxAqPCRr was used to amplify a 134‐bp region of SnToxA. The primer pair Tox3qPCRf and Tox3qPCRr was used to amplify a 143‐bp region of SnTox3. The primer pair 20078‐F and 20078‐R was used to amplify a 189‐bp region of SnTox1. The housekeeping gene actin (*Act1*) was used to normalize gene expression employing the primer pair ActinqPCRf and ActinqPCRf (Tan *et al*., 2008).

For *Py. tritici‐repentis*, *in planta* gene expression analysis was carried out on 2‐week‐old infected Yitpi seedlings (Moffat *et al*., 2014). Lesions were excised from the plant and subjected to RNA isolation, cDNA synthesis and quantitative real‐time PCR as described above. The primer pair PtrPf2F2 and PtrPf2R2 was used to amplify a 108‐bp region of *PtrPf2*. The primer pair ToxAFc and ToxARa was used to amplify a 126‐bp region of *PtrToxA*. The housekeeping gene actin (*Act1*) was used to normalize gene expression employing the primer pair Act1F2 and Act1R2 (150 bp).

*In vitro* gene expression analysis for both fungi was carried out on freeze‐dried mycelia of 3‐day‐old cultures grown in Fries 3 broth. All primer sequences are shown in Table S2 (see Supporting Information).

### Construction of *PnPf2* and *PtrPf2* gene knockout vectors

*Parastagonospora nodorum* SN15 strains carrying deletion in *PnPf2* were created through genetic transformation using a gene knockout vector generated from fusion PCR (Solomon *et al*., 2006b). The primer pair 5_Pf2F and 5_Pf2R was used to amplify a 653‐bp 5′ UTR fragment. This was fused to a phleomycin resistance cassette (*Ble*) amplified from pAN8‐1 using pAN8f and pAN8r. The resulting construct was then fused to an 894‐bp 3′ UTR amplified by the primer pair 3_Pf2F and 3_Pf2R (Fig. S4A). The *PnPf2* knockout vector was then transformed into *P. nodorum* SN15 using polyethyleneglycol (PEG)‐mediated transformation (Solomon *et al*., 2004). Mutants that carry the appropriate gene deletion were identified using PCR (Fig. S4B). A robust quantitative PCR method was used to determine the copy number of the *PnPf2* deletion construct in all transformants to identify appropriate mutants that carry single‐copy integration (Fig. S4C) (Solomon *et al*., 2008). Two gene deletion mutants (*pf2‐63* and *pf2‐69*) and an ectopic strain (Ect) that carry single‐copy integration were retained for further studies.

Similarly, for *Py. tritici‐repentis*, *PtrPf2* was deleted through genetic transformation using a gene knockout vector generated from fusion PCR. The primer pair PtrPf2_5′f and PtrPf2_5′r was used to amplify a 1.3‐kbp 5′ UTR fragment. The primer pair PtrPf2_3′f and PtrPf2_3′r was used to amplify a 1.2‐kbp 3′ UTR fragment. These two fragments were simultaneously fused to a phleomycin resistance cassette (*Ble*) amplified from pAN8‐1 using pAN8f and pAN8r (Fig. S11A). The *PtrPf2* knockout vector was amplified with nested primers and then transformed into *Py. tritici‐repentis* M4 (Moffat *et al*., 2014). Mutants that carry the appropriate gene deletion were identified using a series of PCRs (Fig. S11B, C). Quantitative PCR was used to determine the copy number of the *PnPf2* deletion construct in all transformants to identify appropriate gene deletion mutants that carry single‐copy integration (Fig. S11D) (Moffat *et al*., 2014; Solomon *et al*., 2008). Three gene deletion mutants (*pf2‐a*, *pf2‐b* and *pf2‐c*) and an ectopic strain (E‐1) were retained for further studies.

All PCR amplifications were performed with Phusion Taq DNA polymerase (New England Biolabs, Ipswich, MA, USA).

### Genetic complementation

A 4255‐bp region, containing *PnPf2*, the 982‐bp native promoter and 985‐bp terminator regions, was amplified using Pf2compFGib and Pf2compRGib. The resulting DNA fragment was fused to a 5424‐bp hygromycin resistance cassette (*Hph*) amplified from pAN7‐1 using pAN7Fgib and pAN7Rgib (Fig. S15A, see Supporting Information). The genetic complementation construct *PnPf2‐Hph* was transformed into *P. nodorum pf2‐69*. The transformation procedure produced 35 hygromycin‐resistant transformants. The insert copy number was determined using the robust quantitative PCR method described by Solomon *et al*. (2008) with minor modifications. Briefly, *P. nodorum* MM102 strain containing one predetermined copy of *Hph* was used as a genomic DNA quantitative standard at 20, 6.7, 2 and 0.67 ng/μL. All reactions were performed with a Quantitect SYBR Green RT‐PCR kit (Qiagen) and Bio‐Rad CFX96 system. To determine the copy numbers of the *PnPf2‐Hph* complementation cassette, 6.7 ng/μL of genomic DNA from two transformants, *pf2*::*PnPf2‐17* and *pf2*::*PnPf2‐31*, were used in each quantitative PCR (Fig. S15B). Mutants carrying a single copy will amplify at a *Cq* value similar to that of the MM102 6.7 ng/μL standard. Both transformants contained single‐copy integration based on the relative quantification of the template amount (i.e. *pf2*::*PnPf2‐17*, 6.6 ng/μL; *pf2*::*PnPf2‐31*, 6.3 ng/μL) compared with the MM102 (6.7 ng/μL) genomic standard. *Parastagonospora nodorum pf2*::*PnPf2‐17* was selected for further analysis and is referred to as *pf2*::*PnPf2*.

### *PnPf2* and *SnTox1* double deletion in *P. nodorum*

The *SnTox1* deletion construct was constructed by Tan *et al*. (2015) by replacing the effector gene with the *Hph* cassette (Fig. S8A). This was transformed into *P. nodorum pf2‐69* to facilitate *SnTox1* deletion homologous gene recombination (Solomon *et al*., 2004). A PCR assay was used to select for transformants deleted in *SnTox1* (Fig. S8B). Following this, quantitative PCR was used to determine the insert copy number (Fig. S8C). Mutants that carry single‐copy integration were selected for further analysis.

### Infection assays

Whole‐plant infection assay was performed as described in Solomon *et al*. (2005). Pycnidiospore inoculum was prepared to a concentration of 1 × 10^6^ spores/mL in 0.5% w/v gelatin and sprayed onto 2‐week‐old wheat seedlings using a hand‐held air brush sprayer. Plants were placed in 100% relative humidity at 21 °C, followed by 5 days at 21 °C under a 12‐h photoperiod to facilitate SNB development prior to examination. Disease severity was visually determined. A score of zero indicates no disease symptoms, whereas a score of nine indicates a fully necrotized plant.

A detached leaf assay on benzimidazole agar was used to assess the virulence of all *Py. tritici‐repentis* strains (Benedikz *et al*., 1981; Solomon *et al*., 2004). Small mycelial plug cut‐outs were used as inoculum on detached leaves as conidiospore production was abolished in all *ptrpf2* mutants. Infection was allowed to develop for 7 days prior to examination.

Trypan blue staining of infected wheat leaves was performed as described previously (Solomon *et al*., 2004).

### Genetic mapping and interval QTL analysis

Seedling infection, disease scoring and QTL mapping were performed on a wheat population consisting of 177 DH lines derived from a cross between Calingiri and Wyalkatchem, essentially as described in Phan *et al*. (2016). The DH population was genotyped with diversity array technology and simple sequence repeat markers (Phan *et al*., 2016).

## Supporting information

Additional Supporting Information may be found in the online version of this article at the publisher's website:

**Table S1** Quantification of conidiospores from *Pyrenophora tritici‐repentis* M4 wild‐type, E‐1 ectopic and *PtrPf2* deletion strains grown on V8‐PDA solid medium for 7 days.

**Table S2** Primers used throughout this study.

**Data S1** Amino acid sequences used to construct a bootstrap consensus phylogenetic tree (Fig. 1).

**Fig. S1** Scaled schematics of the CD‐Blast conserved domain structure for SNOG_00649 (PnPf2), M4_04272 (PtrPf2), M4_11517 and AbPf2. Amino acid numbers are shown. The GAL4‐like Zn_2_Cys_6_ binuclear cluster domain is indicated in orange and the fungal transcription factor regulatory middle homology region is indicated in blue.

**Fig. S2** Nucleotide alignment between M4_04272 and PTRG_06982 (A) and M4_11517 and PTRG_11777 (B). Start (blue) and stop (red) codons are indicated.

**Fig. S3** Nucleotide alignment of the putative promoter region of ToxA (yellow) from *Parastagonospora nodorum* (SN15 and SN4) and *Pyrenophora tritici‐repentis* (M4 and Pt‐1C‐BFP).

**Fig. S4** Construction of the *PnPf2* knockout vector. (A) The 5′ and 3′ untranslated regions (UTRs) of *PnPf2* were polymerase chain reaction (PCR) amplified (i) and fused to *Ble* to give the *PnPf2* knockout vector (ii). (iii) The vector was transformed into SN15 to facilitate gene knockout via homologous recombination of the 5′ and 3′ flanks. (B) Five knockout mutants (*pf2–*) and two ectopic strains (*Pf2–*) were selected from PCR screening using 3_00649ScrF/R for insert copy number determination using quantitative PCR. (C) All possess single‐copy integration, except for *pf2‐19*. Consequently, *pf2‐63*, *pf2‐69* and *Pf2‐18* (Ect) were selected for further studies.

**Fig. S5** Assessment of colony morphology (A) and pycnidiospore production (B) on 2‐week‐old Petri dish fungal cultures grown on V8‐PDA. Error bars are shown as standard error of the mean. The experiment was performed in biological replicates (*n* = 3).

**Fig. S6** Whole‐plant virulence assay of *Parastagonospora nodorum* strains on wheat cv. Chinese Spring (*tsn1, Snn1*, *snn3*) (A) and Wyalkatchem (*tsn1*, *snn1, Snn3*) (B).

**Fig. S7** Genetic complementation of *pf2‐69* restores virulence on BG261 and BG220.

**Fig. S8** *SnTox1* deletion in *pf2‐69*. (A) The *SnTox1‐Hph* gene knockout cassette was polymerase chain reaction (PCR) amplified from *Parastagonospora nodorum tox1‐6* (i). This was used to delete *SnTox1* in *pf2‐69* (ii), resulting in mutants that lacked *PnPf2* and *SnTox1* (iii). (B) PCR using the primer pair 20078ScreenF/R was used to screen for *SnTox1* deletion in all transformants. Mutants deleted in SnTox1 produced a 4.9‐kb PCR amplicon. PCR amplification of ectopic transformants resulted in a 2.6‐kb band. (C) One ectopic and three *tox1* mutants were analysed for insert copy number using quantitative PCR. All strains possess single‐copy integration.

**Fig. S9** The distribution of SN15, *pf2‐tox1‐6* and *tox1‐6* whole‐plant spray disease severity scores on the Calingiri × Wyalkatchem DH population at the seedling stage. Data from SN15 and *tox1‐6* were derived from Phan *et al*. (2016).

**Fig. S10** A genetic map of chromosomes with genetic markers on the right and the centimorgan (cM) distances between loci on the left. Quantitative trait loci (QTLs) associated with *pf2‐tox1‐6* infection are indicated in purple.

**Fig. S11** Construction of the *PtrPf2* knockout vector. (A) The 5′ and 3′ untranslated region (UTR) of *PtrPf2* was polymerase chain reaction (PCR) amplified (i) and fused to *Ble* to give the *PtrPf2* knockout vector (ii). This was amplified with the nested primer pair PtrPf2Nf/PtrPf2Nr and transformed into the *Pyrenophora tritici‐repentis* M4 wild‐type to facilitate gene knockout (iii). (B) *PtrPf2*‐specific amplification using the primer pair PtrPf2Sf and PtrPf2Sr identified three knockout (*pf2–*) and one ectopic (E‐1) mutant. (C) Gene disruption of the *PtrPf2* locus was confirmed using the primer pair PtrPf2_5′f Phleo5 which amplifies a 1.4‐kb fragment in strains that carry the appropriate gene deletion. (D) Transformants were analysed for insert copy number using quantitative PCR. All strains possess single‐copy integration except for E‐1. *Pyrenophora tritici‐repentis* is very difficult to transform. Consequently, we were only able to identify one ectopic mutant. However, double integration did not reduce the level of fitness in the E‐1 strain.

**Fig. S12** Virulence of *Pyrenophora tritici‐repentis* M4 wild‐type, E‐1 and *PtrPf2* deletion mutants on wheat cv. Yitpi (*Tsn1*).

**Fig. S13.** Colony morphology of the *P. tritici‐repentis* M4 wild‐type, E‐1 ectopic and *PtrPf2* deletion strains on V8‐PDA.

**Fig. S14** Whole‐plant virulence assay of *Parastagonospora nodorum* strains on wheat cv. BG223.

**Fig. S15** Genetic complementation of *Parastagonospora nodorum pf2‐69*. (a) Fusion polymerase chain reaction (PCR) was used to construct the *PnPf2‐Hph* gene complementation vector. (b) *PnPf2‐Hph* insert copy number determination using quantitative real‐time PCR. Biological triplicates were used in the copy number assay. Error bars are shown as the standard error of the mean.
