# Supplementary material for: A functionally conserved Zn2Cys6 binuclear cluster transcription factor class regulates necrotrophic effector gene expression and host‐specific virulence of two major Pleosporales fungal pathogens of wheat
Source: Mol Plant Pathol. 2017 Jan 24;18(3):420–34. doi: 10.1111/mpp.12511 (PMC6638278; doi:10.1111/mpp.12511)
Supplement: Supplementary file 1 — Table S1 Quantification of conidiospores from Pyrenophora tritici‐repentis M4 wild‐type, E‐1 ectopic and PtrPf2 deletion strains grown on V8‐PDA solid medium for 7 days. [file MPP-18-420-s001.docx]

|  |  |  |
| --- | --- | --- |
|  | **Replicate** | **Conidiospores/plate** |
| M4 | 1 | 6300 |
|  | 2 | 3400 |
|  | 3 | 5500 |
| E-1 | 1 | 5950 |
|  | 2 | 5450 |
|  | 3 | 4600 |
| *pf2-a* | 1 | 0 |
|  | 2 | 0 |
|  | 3 | 0 |
| *pf2-b* | 1 | 0 |
|  | 2 | 0 |
|  | 3 | 0 |
| *pf2-c* | 1 | 0 |
|  | 2 | 0 |
|  | 3 | 0 |
|  |  |  |
