# Supplementary material for: A functionally conserved Zn2Cys6 binuclear cluster transcription factor class regulates necrotrophic effector gene expression and host‐specific virulence of two major Pleosporales fungal pathogens of wheat
Source: Mol Plant Pathol. 2017 Jan 24;18(3):420–34. doi: 10.1111/mpp.12511 (PMC6638278; doi:10.1111/mpp.12511)
Supplement: Supplementary file 2 — Table S2 Primers used throughout this study. [file MPP-18-420-s002.docx]

| **Primer** | **Sequence (5’ - 3’)** |
| --- | --- |
| 20078-F | TCCAGCAACAGCACATCA |
| 20078-R | TCCTGGAGTATGGCAAATTG |
| 3_00649ScrF | TGACTATGAAAATTCCGTCACC |
| 3_00649ScrR | GATCGCTTCAGGAACAAAGC |
| 5_Pf2F | GCTGCAGAGTCGTTTCAACC |
| 5_Pf2R | TGTGACTTTTGGTTACGCCGTCTCTTAACAGGAGCGGATGTGG |
| 3_Pf2F | TCTCCTATGAGTCGTTTACCCAGAACCTAGATTCAGCCTCCAAGG |
| 3_Pf2R | TAACACGTCCTTTGTGAACG |
| ActinqPCRf | AGTCGAAGCGTGGTATCCT |
| ActinqPCRr | ACTTGGGGTTGATGGAG |
| pAN7FGib | ACTTGAGCCTAAAATCCGCC |
| pAN7RGib | CCTGTGTGTAGAGATACAAGGG |
| pAN8f | AGACGGCGTAACCAAAAGTCACA |
| pAN8r | TTCTGGGTAAACGACTCATAGGAGA |
| Pf2compFGib | CCCTTGTATCTCTACACACAGGGCGGCCGCTTAAACATCAGGGCAGCGC |
| Pf2compRGib | GGCGGATTTTAGGCTCAAGTAGACTGAGCATGAAAGAGCC |
| Pf2qPCRf | CATTCATCAGTCTCTGGAACCG |
| Pf2qPCRr | CGAATCTCGACGCCTTGGG |
| Phleo5 | CTCCGTCTTCCGTAGCCGTG |
| PtrPf2_5'f | TCTTATTCGCGGTCTCGACG |
| PtrPf2_5'r | TGTGACTTTTGGTTACGCCGTCTGAAAGCCCAAGCGTCCAAAG |
| PtrPf2_3'f | TCTCCTATGAGTCGTTTACCCAGAAATGCAGAGTCACCACATGGG |
| Ptrpf2_3'r | GGAAAGCCAAAAAGTCGCGT |
| PtrPf2Sf | CCCGCTCGATGTATCACGAA |
| PtrPf2Sr | GGCGTGGACGTCAGAGATAG |
| ToxAqPCRf | CGATCCCGGTTACGAAAT |
| ToxAqPCRr | TTGACATGCAGCTTCCCT |
| Tox3qPCRf | AATGTCGACCGTTTTGACC |
| Tox3qPCRr | GGTTGCCGCAGTTGATATAA |
| PtrPf2F2 | CCATGATCTCCGCACCTACC |
| PtrPf2R2 | TCTCGTAAGGACCGGGAGTT |
| Act1F2 | AGACCTTCAACGCTCCCGCC |
| Act1R2 | TGGCGTGGGGAAGAGCGAAAC |
| ToxAFc | TAAACGCCGATACAGTGCGA |
| ToxARa | AAAGCTCATAAACGTCCCCC |
| 20078KO5’f | TAGCCGTAGGAGTCCTAATTTAGC |
| 20078KO3’r | GCTAGACAACCAATACCTGCTG |
| 20078ScreenF | ATGATGCATAGAAGGGTACCAC |
| 20078ScreenR | GCAGAAGGGAAAGCAACAA |
