# Supplementary material for: A functionally conserved Zn2Cys6 binuclear cluster transcription factor class regulates necrotrophic effector gene expression and host‐specific virulence of two major Pleosporales fungal pathogens of wheat
Source: Mol Plant Pathol. 2017 Jan 24;18(3):420–34. doi: 10.1111/mpp.12511 (PMC6638278; doi:10.1111/mpp.12511)
Supplement: Supplementary file 3 — Data S1 Amino acid sequences used to construct a bootstrap consensus phylogenetic tree (Fig. 1). [file MPP-18-420-s003.docx]

**Sc EDV09978.1**

MTLTKQTCAKQACDCCRIRRVKCDGKRPCSSCLQNSLDCTYLQPSRKRGPKSIRLRSLKRIAEVQRESGPNTIATAPVIYKRVPKKLIDQCLRLYHDNLYVIWPLLSYDDLHKLLEEKYNDNYVYWFLTALSAATLSDLQTEIKSEEEVTFTGKQLSNLCISSCQQFDDLDNSNIFNIMTYYCLHRSFAQISNARTSYRLCCEAVGLITVAGLHREETYGSLTFEEQQLRRKLYYLLLMTERYYAIYLHCATSLDATIAPPQLELVTDPQLSMDSFLEMIRIFTVLGKCFFDALAADSTDASCTEESLKKIWNELHTTSSEIEPWSNGYIDISFSRRWIRILAWKLAYQMRGSNFSLNANNGQIPIEIARDMLIDTYLTPENLYDVHGPGVPVKTLEIATALVDIVGQYDHNMKLEAWNVLHDVCKFAFSLNHYNNDMLKRFSTKCQNALITLPISKPLQLDGYPKNNEDIDP

**Pn SNOG_00649 (SN15)**

MSSSSTTSAPVKRACDSCHRRKVKCIGEGTNPCKNCLSAGLACTYNAIPQKKGPKGSRAKVLSELRENQRNAQLAAGFPPDVGYDGRTLSTTFARAQGLLPNGLVDTCLDFFFANVYPSTPVLHRQKAQELAVNMERSTEAYCLIVSLCAYVMIHANMKVPSNMFSRPEVAQMSNMTLGHALLEESVRVRNGYDFRENPTHITVLTSWFFSGSYFGLARENTAWGYLREATTQAQLLGMHDEETYKHDPLDISRKRVLYWVLFIAERQFALHKHRPISLYPTIHPPSLDEVPSDRPIAVGLELLITLYKNIDDTFISLWNRVHTHTNPAWFSQLHTQLSEAVPAYLECTEAQGVEIRITQQWLKAMAWQLCVCQGLVSSVTNDNCMTFKYPIEISRDLLTMTHQFSQQAMEVHGAELIEKLFDIACCLADVVAVTSFSPDAFALGPRDYVSRFLTLISTLRGGHSRYLPLLLAKLSEVLPNLPLPRSLNLPQTLPASTISMSGTGTVPSNITDDYSAMPATSSPSYPSSELIRRLAAQTGAQLPFGTSQQSMIQAPSSHVEDLSLYDTAHSHSASHSSSSAPRSNSTTPGPYESTMSQRSSQILSHQSVQIPTSHPQHNHMQSHHISVAQTAYDPRFSLQGYPVDPSMMFKQ

**Pn SNOG_00649 (SN79-1087)**

MSSSSTTSAPVKRACDSCHRRKVKCIGEGTNPCKNCLSAGLACTYNAIPQKKGPKGSRAKVLSELRENQRNAQLAAGFPPDVGYDGRTLSTTFARAQGLLPNGLVDTCLDFFFANVYPSTPVLHRQKAQELAVNMERSTEAYCLIVSLCAYVMIHANMKVPSNMFSRPEVAQMSNMTLGHALLEESVRVRNGYDFRENPTHITVLTSWFFSGSYFGLARENTAWGYLREATTQAQLLGMHDEETYKHDPLDISRKRVLYWVLFIAERQFALHKHRPISLYPTIHPPSLDEVPSDRPIAVGLELLITLYKNIDDTFISLWNRVHTHTNPAWFSQLHTQLSEAVPAYLECTEAQGVEIRITQQWLKAMAWQLCVCQGLVSSVTNDNCMTFKYPIEISRDLLTMTHQFSQQAMEVHGAELIEKLFDIACCLADVVAVTSFSPDAFALGPRDYVSRFLTLISTLRGGHSRYLPLLLAKLSEVLPNLPLPRSLNLPQTLPASTISMSGTGTVPSNITDDYSAMPATSSPSYPSSELIRRLAAQTGAQLPFGTSQQSMIQAPSSHVEDLSLYDTAHSHSASHSSSSAPRSNSTTPGPYESTMSQRSSQILSHQSVQIPTSHPQHNHMQSHHISVAQTAYDPRFSLQGYPVDPSMMFKQ

**AbPf2_AFQ61041.1**

MASTPTTTAPVKRACDSCHRRKVKCIGEGTAPCKNCVSAGLACTYNAIPQKKGPKGSRAKVLSELRETQRNAQLAAGFPTDLGFDGRSLSSTFARTPGLMPPALVESCIEYFFAHVYPSEPVLHRQRAQEAAVGMDRSTEAYCMVVALCAYVMIQANHKPPVSVLPRSEMAHMSNVGIGHVLLEESVRVRQGYDHRENPTHYSVLTSWFYSGCYFGLARENTAWSYLRDATTQAQLLGMHDEETYKHDPLDISRKRVLYWLLFIAERTFALHKHRPISLHPTIHPPSLEEVPSDRPIAVGLELMINMFKIIDDTFINLWNRVHNTHASAAWITQVQTQLSEAVPAYFECTEVQEVQIRITQQWLRSQAWQLSVCQGLVSSVSNDIPLTFKYPIEIARDLLTISHQFSQQAMEMHGVGLIEKLFDIACCLTDVVACTSFSPESFALGPRDYVSRFLTLISTLRGGQTRYLPLLLAKLSEVLPNLPLPRSLNLPQTVPTGTLGLNATGSGTLPSNVADDYSANSNATSPSYPSNDLIRRLAAQTGAQLPFNTTQHSMVNVPTTHVGDMSLYDSSHSTAHSHTSSSAPRSTSTTPGPYETPMSQRGSQIVGHPTMQLPTSHGHMQSHHMGVNATAYDPRFSIQSYPVDPMMYKQ

**Bc XP_001547137.1**

MSSSSSVKRACDACHRRKVKCDGINPCRNCSSAQLSCTYHAIPQKKGPKGSRAKVISELRQNQQSSLSAKTANRINGVQSPPCSPTLAPTPGLLTTEMIKECTEFFFANMYPTMPILHRERLEQQALYVDQNVDTYCLLTAMCAFMMIQPGMGIPGDPLGLDNLPGANIVSGTLLLEETLKVRKSYDYCESPTQNSLVTSFFIFACYYGLDLHNKAWFHLREATTLAHTLEMVKEETYHQFDIIESSRRRRLYWLLFVTERSYALQRHRPLSLQATINLPSINDDPTDPQNHHLHGFIHLVNVYRPFDDQFVALWNKTRTDCSPAYLSSLQKQLSEALPVYLNTTENQTADLRTSQQWLRTMVWQLSIQNGCLSSNCDDPSMTFQYPVDIARDLAQMTSQFSHQSMEVHGVGLIEKLFDVACSLTDVLSLLPAKSDPFTMGPRDYLHQFMTMLSILRNGDHRFLPLLLNKVHDVLPGLVNPMLQNAPENTQMCADVDLFDGFGNTGISIPPQNYNTMATSGPNNYKMAHTNGGLAFDKRIEELSSPSVTGSMSAETTPFTSPPIMNPGMEYQALDYPGFHDLNSHAHGMDFKREFGVENGENSFGVSSPGGMSEMSSGVASVNGNGIGGRRPPIRQGSGSSYGMPLPRSIPEFNHGHFSRASTGGEDMGVGVNGVDLSHHGYR

**Ptr M4_04272 (M4)**

MASNPTTTAPVKRACDSCHRRKVKCIGEGTAPCKNCVSAGLACTYNAIPQKKGPKGSRAKVLSELRETQRNAQLAAGFPTDLGYDGRSLPSTFARTQGLLPPALVESCIEYFFNNVYPTEPVLHRQRAQEAAVGMDRSTEAYCMIVALCAYVMIQGNHKPHVNVLPRQEMAHMSNVGIGHVLLEESVRVRQGYDHRENPTHMSVLTSYFYHGCYFGLARENTAWTYLREATTQAQLLGMHDEETYKHDPLDVSRKRVLYWLLFIAERTYALHKRRPITLHPTIHTPSLDEVPSDRPIAVGLELMINMFKIIDDNFINLWNRVHNTHASAAWIAQVQTQLSEAVPAYFECTEAQEVQIRITQHWLRSQAWQLSVSQGLVSSVTNDPPLTFKYPIEISRDLLTTSHQFSQQAMEVHGVGLIEKLFDIACCLTDVVACTTFSPDQFALGPRDYVSRFLTLISTLRGGQSRYLPLLLSKLNEVLPNLPLPRSLNLPQTVSASTLGLNASGSGTLPSNVADDFSASPASSSPSYPSNDLIRRIAAHTGAQLPFNTTQQSMISAPTTHVGDLSLYDTSQQSTTHSSSSGPRSNSTTPGPYETPLSQSRSQIVGHSAMQLPTSHGHMQSHHMGVNATAYDPRFSIQNYPVDPMMYKH

**Ptr M4_11517 (M4)**

MDRSTEAYCMIVALCAYVIIQANHKPHANVLPRQEITRMFNVGIGHVLLEESVRCPDLVFYYGCYLGLALKNTALTYLREATTQAQLLGMHDEETYKYDLLDESPKRALYWLLSIAERTCALHTRRSITLHPLMHLTSLDEVPSDQLITIYLELIINMFKIIDDGCIKLSKGVHETNESTAWILQVQTRLSEAVPADLECTEAQEVQIRTTQQWLMLSIWELGVNQGLVNEVNKDPSLTYICPIDISRELLTTLQQFPKQVMQVHGVGLVST

**Ptr XP_001937314.1 (Pt-1C-BFP)**

MTQGTFKDISPNAGATLDDETSHTSDLFSFHGFQSYHDCSGQAGLRQNCVSAGLACTYNAIPQKKGPKGSRAKVLSELRETQRNAQLAAGFPTDLGYDGRSLPSTFARTQGLLPPALVESCIEYFFNNVYPTEPVLHRQRAQEAAVGMDRSTEAYCMIVALCAYVMIQGNHKPHVNVLPRQEMAHMSNVGIGHVLLEESVRVRQGYDHRENPTHMSVLTSYFYHGCYFGLARENTAWTYLREATTQAQLLGMHDEETYKHDPLDVSRKRVLYWLLFIAERTYALHKRRPITLHPTIHTPSLDEVPSDRPIAVGLELMINMFKIIDDNFINLWNRVHNTHASAAWIAQVQTQLSEAVPAYFECTEAQEVQIRITQHWLRSQAWQLSVSQGLVSSVTNDPPLTFKYPIEISRDLLTTSHQFSQQAMEVHGVGLIEKLFDIACCLTDVVACTTFSPDQFALGPRDYVSRFLTLISTLRGGQSRYLPLLLSKLNEVLPNLPLPRSLNLPQTVSASTLGLNASGSGTLPSNVADDFSASPASSSPSYPSNDLIRRIAAHTGAQLPFNTTQQSMISAPTTHVGDLSLYDTSQQSTTHSSSSGPRSNSTTPGPYETPLSQSRSQIVGHSAMQLPTSHGHMQSHHMGVNATAYDPRFSIQNYPVDPMMYKH

**Ptr XP_001942108.1 (Pt-1C-BFP)**

MVFGRLGFLIAQAYFVGIGDHLARLECTYDAIPQKKGPKGSRAKVLSKLHKTQRNTQLAAGFPTDLRCDGLSLPSTFARTQGLLPPALVESCIEYFFNNFYPTEPVLQHQKAQEAVDGMDRSTEAYCMIVALCAYVIIQANHKPHANVLPRQEITRMFNVGIGHVLLEESCPDLVFYYGCYLGLALKNTALTYLREATTQAQLLGMHDEETYKYDLLDESPKRALYWLLSIAERTCALHTRRSITLHPLMHLTSLDEVPSDQLITIYLELIINMFKIIDDGCIKLSKGVHETNESTAWILQVQTRLSEAVPADLECTEAQEVQIRTTQQWLMLSIWELGVNQGLVNEVNKDPSLTYICPIDISRELLTTLQQFPKQVMQVHGVGLVST

**Bgh KFY34063.1**

MSTSVKRACDACHRRKVKCDGINPCRNCSSAQLSCTYNAIPQKKGPKGSRAKVISELRETQRQSSLSSKVTSRMTGVCPVTYNASLAQTPGLLTNDLIRECVDFFFTNMYPTLPILHRGRLEQQILYAEKNVDTYCLLTSLCAFMMIQPGMGIPGAPLRAENLPDANLVSGNLLMQETLRIRKSYDYLEVPTLNSLATSFFLFGCYFGLNLHNNAWFHLREATTLAHVNGLQKEETYSQFDVADASRRRRLYWLLFVTERAYALQRHRPLSLQATISPPSQNDDPTDPQACHLNGFIDLVSLFLPFDEALVTLWNKTSTECSPNYLVALQKDLADALPAYLSSNENQTADLRTSQQWLRTMVWQLSMQNGCLSSAHEEPSMTFQYPVDISRDLISMTSQFSHQSMDIHGIGLIEKLFDIACSLTDILTLLPPNLYSFTLGPQDYLHDFIGLLAVLRHGNHRFLPLLLAKVNDVLPRLVNPMLRAVPDPPPDVCNEIDVFDGYDTAGGSITMASRFPEYAGDVNSGSPDFKIEPGDYEGTNVGHGLPAFEQQSMDELGSPVSSSLGGTSSSSEHGAPSYSVDVRSPVEYSYSLQEVGSRSSSYVDELPGSKSEYNRAVETFKRPVLRSCSGSPTIGANTGSSGGNSVSSGSSGSCATACAINTGPSAATPTVAGGNGPVSSGFAMQIPRSVPKQVYPMARSLSSTCGGEASLR

**Bgt EPQ63060.1**

MSTSVKRACDACHRRKVKCDGINPCRNCSSAQLSCTYNAIPQKKGPKGSRAKVISELRETQRQSSLSSKVTSRITGVCPVTYNASLAPTPGLLTNDLIRDCVDFFFTNMYPTLPILHRGRLEQQILYAEKNVDTYCLLTSLCAFMMIQPGMGIPGAPLRAENLPDANLVSGNLLMQETLRIRKSYDYLEVPTLNSLATSFFLFGCYFGLNLHNNAWFHLREATTLAHVNGLQKEETYSQFDVADASRRRRLYWLLFVTERAYALQRHRPLSLQATISPPSQNDDPTDPQACHLNGFIDLVSLFLPFDEALVTLWNKTSTECSTNYLAALQKDLADALPAYLTSNENQTADLRTSQQWLRTMVWQLSMQNGCLSSAHEEPSMTFQYPVDISRDLISMTSQFSHQSMDIHGIGLIEKLFDIACSLTDVLTLLPPNLYSFTLGPQDYLHDFIGLLAVLRHGNHRFLPLLLAKVNDVLPRLVNPMLRAVPDPPPDMCNEIDVFDGYDTAGGSITMASRFPEYAGDVNSGSPEFKIEPGDYEGTNVGHGLPAFEQQNMDELGSPVSSSLGGTSSSSEHGATSYSVDVRSPVEYPYSLQEVGSRSSSYVDELPGSKSEYNRAVETFKRPILRSCSGSPTIGANTGSSGGNSVSSGSSGSSATTCAISVGPSAATPTVAGGNGPVSNGFAMQIPRSVPKQVYPMARSLSSTCGGEASLR

**Cc XP_007706970.1**

MASNPTSTAPVKRACDSCHRRKVKCIGEGTAPCKNCVSAGLACTYNAIPQKKGPKGSRAKVLSELRETQRNAQLAAGYPTDLGFDGRSLPATFARTQGLLPPALVESCIEYFFNHVYPSEPVLHRQRAQEAAVGMDRSTESYCMIVALCAYVMIQANHKPPVSVLPRPEMAHMSNVGVGHILLEESVRVRQGYDHRENPTHTSVLTSWFYSGCYFGLARENTAWTYLREATTHAQLLGMHDEETYKHDPLDVSRKRVLYWLLFIAERTYALHKHRPITLHPTIHPPQLDEVPTDRPIAVGLELMINMFKIIDDTFINLWNRVHNTHVSAAWITQVQTQLSEAVPAYFECSEAQEVQIRITQQWLKSQTWQLSVCQGLVSSVSNDVPLTFKYPIEIARDLLTISHQFSQQSMEVHGVGLIEKLFDIACCLTDVVACTSFSPDTFALGPRDYVSRFLTLISTLRGGQSRYLPLLLAKLSEVLPNLPLPRSLNLPQTVPASTLGLNATGSGTLPSNVAEDFSASPASSSPSYHSNDLIRRLAAQTGAQLPFNTTQQSMIPAPTTHVGDLSLYDTSHNTSHSTSTANRSTSTTPGPYEAPMSQSRSQIVGHSNMQIPTSHSHMQNHQMGVNATGYDPRFSIQNYPVDPMMYKQ

**Cgl EQB44756.1**

MTQAVKRACDACHRRKVKCDGVNPCRNCHTAQLSCTYNAIPQKKGPKGSRAKVISELRETQRQTSLSAKVQNRMNGMSGPPGPVSLAPTPGLLSSELVKECIDFYFANLYEKMPILDRHKIEQQLPYMERDPDTYCLLTSLCAFMMLQPGMSMPAGDPFNLDVMPGAAIVATQLLLEETLQVRKGYDYLTGPSEGPTFNTFATNFFIYGCYHGMEMHNKAWYYLREASTMMHMSGMTKEETYRSPQWDNAESSRRRRLYWLFYVMERAHAIQHQRPLTLQATIQPPNSADDPSDPLSHQLNSFIMLVNLFHPFDDAFTAVWNKTRSNLYVSNLQKQLNEVLPTYLCQDGQLSDLRTNQQWLKTTVWQLTHGSMNSQNEESMNNFQYPVDLSREMLMNLASHFPGQGMELMGSGLIEKLFQITCNMTDFLAVQPAARDPFTVGPREQLNQTLNIVAVLRHSDFRFLPLLLNKVADIMPRLTNPMLQNAPENANLGAIDIFDGFGNAGMAQPPQMQMQMDTDYDRKFSVADYDKKYGMSDMNSNVSDGNVSTGAPSVSVPATTADMNSPFASSPAIMSPPMEYPHNGMNDYGCAQMPDMVMSPMGQNGSSIGGSNQHQQHQSHHMLQNQGMNQQQMQPSMNQHHNHNMQQGQNMGSISPPSFQGQQQMNAPSIPTSHPMGPGGNNLMNSISRQQPQRANSYAIPQQPLPRTVGETFHALQRANSDMPGMTGMPTEMDFNTLR

**Cgr EFQ26001.1**

MTQAVKRACDACHRRKVKCDGVNPCRNCHTAQLSCTYNAIPQKKGPKGSRAKVISELRETQRQTSLSAKVQNRMNGIPNPPGPVTLSPTPGLLSSELVKECIDFYFANLYEKIPILDRRQIDLQLPYMEQNRDTYCLMTSLCAFMLLQPGMSMPTGDPYNLDVMPGATIVASQLLLDETLQVRKGYDYLDSLSFNTLATNFFIYGCYHGLELHDKAWFYLREVSTMMHLNGMSKEETYHGPQWENAESSRRRRLYWLFYIMERAHAIQRQRPLTLQATINPPNSADDPSDPLSHQLNSFIMLVNLFRPFDDAFTAVWNKTRTNLYVANLQKQLNEVLPTYLCQDGQLSDLRTNQQWLKSTVWQLTHGNMNSQNEDNMNFNYPVDLSRELLMTLASHFPGQGMELMGSGLIEKLFEISCNMTDFLAVQPAARDPFTVGPREQLNQTLNIVAVLRHGDHRFLPLLLNKVAEIMPRLTNPMLQNAPENSNLGNMDIFDGFGNAGMAQPPPQMQMQMDTEYERKYAATDYDKKYGMSDMNSNGSDTNTIAASSAGAPSVPPATTADMNSPFASSPPIMSPPMEYPRNGMNDYGCTQMPDMVMSPMGQNGSAMNGSSTMSNQHRQQQSHHLIQNQCMTPQQMQSSIGQHQNMQQGNITPPSIQGQQGLNSSSIPTSHPLGPGGNNLMNSIPRQQPQRATSFAIQQPVPRTVGDFHALQRANSDMPGLSGIGTEMDFNTLR

**Ch EMD86706.1**

MAENHTKALAKRACDRKVKCVPGITDCKNCNSAGLSCTYNTIPQQKGPSKGSRGKVLRELRKNQPDDQLAAGHSTALNCGGYSNPATLDHTQGLLLPLALINSCIEYFFSHVYFSEPVLHRERALAAVYYMESFTESYCIIAALCAYVIVKANYNPPTNVLSLEMAHMSNFDIGQRLLEESVRERQGYDHQKAPTHMSVLTSWFYSGCYFGLEQEDKAWMYLQEAIRYAQILGMHNEETYKHDSLDAPETRALYWLLFIAERTYALRKHRRVTLHPTINLPQSAGMTFGPFISVRLNIMINMFQIIDNTVIDLRHKTAISVNDVYILDLHKCLSEAVPASFERYEAQICITQQWLRLQIWELSFRHKVLSSVSKDAPLTFEYPITIARDLLAIFNQFSQQFIDVYRVDLIEKLVKIACCLTDIDKFYSTEPFVRDVQLEFLKLIWKLRGGQSLYHHLLLDELRIAESPKSTFYNTARDP

**Ch EMD89852.1**

MASNPTSTAPVKRACDSCHRRKVKCIGEGTAPCKNCVSAGLACTYNAIPQKKGPKGSRAKVLSELRETQRNAQLAAGYPTDLGFDGRSLPATFARTQGLLPPALVESCIEYFFNHVYPSEPVLHRQRAQEAAVGMDRSTESYCMIVALCAYVMIQANHKPSVSVLPRPEMAHMSNVGVGHILLEESVRVRQGYDHRENPTHTSVLTSWFYSGCYFGLARENTAWTYLREATTHAQLLGMHDEETYKHDPLDVSRKRVLYWLLFIAERTYALHKHRPITLHPTIHPPQLDEVPTDRPIAVGLELMINMFKIIDDTFINLWNRVHNTHVSAAWITQVQTQLSEAVPAYFECSEAQEVQIRITQQWLKSQTWQLSVCQGLVSSVSNDVPLTFKYPIEIARDLLTISHQFSQQSMEVHGVGLIEKLFDIACCLADVVACTSFSPDAFALGPRDYVSRFLTLISTLRGGQSRYLPLLLAKLSEVLPNLPLPRSLNLPQTVPASTLGLNATGSGTLPSNVAEDFSGSPASTSPSYHSNDLIRRLAAQTGAQLPFNTTQQSMIPAPTTHVGDLSLYDTSHSTSHSTSTANRSTSTTPGPYEAPMSQSRSQIVGHSNMQIPTSHSHMQNHQMGVNATGYDPRFSIQNYPVDPMMYKQ

**Cm XP_007692222.1**

MASNPTSTAPVKRACDSCHRRKVKCIGEGTAPCKNCVSAGLACTYNAIPQKKGPKGSRAKVLSELRETQRNAQLAAGYPTDLGFDGRSLPATFARTQGLLPPALVESCIEYFFNHVYPSEPVLHRQRAQEAAVGMDRSTESYCMIVALCAYVMIQANHKPPVSVLPRPEMAHMSNVGVGHILLEESVRVRQGYDHRENPTHTSVLTSWFYSGCYFGLARENTAWTYLREATTHAQLLGMHDEETYKHDPLDVSRKRVLYWLLFIAERTYALHKHRPITLHPTIHPPQLDEVPTDRPIAVGLELMINMFKIIDDTFINLWNRVHNTHVSAAWITQVQTQLSEAVPAYFECSEAQEVQIRITQQWLKSQTWQLSVCQGLVSSVSNDVPLTFKYPIEIARDLLTISHQFSQQSMEVHGVGLIEKLFDIACCLTDVVACTSFSPDAFALGPRDYVSRFLTLISTLRGGQSRYLPLLLAKLSEVLPNLPLPRSLNLPQTVPASTLGLNATGSGTLPSNVAEDFSASPASSSPSYHSNDLIRRLAAQTGAQLPFNTTQQSMIPAPTTHVGDLSLYDTSHNTSHSTSTANRSTSTTPGPYEAPMSQSRSQIVGHSNMQIPTSHSHMQNHQMGVNATGYDPRFSIQNYPVDPMMYKQ

**Cs XP_007698262.1**

MASNPTSTAPVKRACDSCHRRKVKCIGEGTAPCKNCVSAGLACTYNAIPQKKGPKGSRAKVLSELRETQRNAQLAAGYPTDLGFDGRSLPATFARTQGLLPPALVESCIEYFFNHVYPSEPVLHRQRAQEAAVGMDRSTELYCMIVALCAYVMIQANHKPPVSVLPRPEMAHMSNVGVGHILLEESVRVRQGYDHRENPTHTSVLTSWFYSGCYFGLARENTAWTYLREATTHAQLLGMHDEETYKHDPLDVSRKRVLYWLLFIAERTYALHKHRPITLHPTIHPPQLDEVPTDRPIAVGLELMINMFKIIDDTFINLWNRVHNTHVSAAWITQVQAQLTEAVPAYFECSEAQEVQIRITQQWLKSQTWQLSVCQGLVSSVSNDVPLTFKYPIEIARDLLTISHQFSQQSMEIHGVGLIEKLFDIACCLTDVVACTSFSPDAFALGPRDYVSRFLTLISTLRGGQSRYLPLLLAKLSEVLPNLPLPRSLNLPQTVPASTLGLNATGSGTLPSNVAEDFSASPASSSPSYHSNDLIRRLAAQTGAQLPFNTTQQSMIPAPTTHVGDLSLYDTSHNTSHSTSTANRSTSTTPGPYEAPMSQSRSQIVGHSNMQIPTSHSHMQNHQMGVNATGYDPRFSIQNYPVDPMMYKQ

**Cv EUN27933.1**

MASNPTSTAPVKRACDSCHRRKVKCIGEGTAPCKNCVSAGLACTYNAIPQKKGPKGSRAKVLSELRETQRNAQLAAGYPTDLGFDGRSLPATFARTQGLLPPALVESCIEYFFNHVYPSEPVLHRQRAQEAAVGMDRSTESYCMIVALCAYVMIQANHKPPVSVLPRPEMAHMSNVGVGHILLEESVRVRQGYDHRENPTHTSVLTSWFYSGCYFGLARENTAWTYLREATTHAQLLGMHDEETYKHDPLDVSRKRVLYWLLFIAERTYALHKHRPITLHPTIHPPQLDEVPTDRPIAVGLELMINMFKIIDDTFINLWNRVHNTHVSAAWITQVQTQLSEAVPAYFECSEAQEVQIRITQQWLKSQTWQLSVCQGLVSSVANDVPLTFKYPIEIARDLLTISHQFSQQSMEVHGVGLIEKLFDIACCLTDVVACTSFSPDAFALGPRDYVSRFLTLISTLRGGQSRYLPLLLAKLSEVLPNLPLPRSLNLPQTVPASTLGLNATGSGTLPSNVAEDFSASPASSSPSYHSNDLIRRLAAQTGAQLPFNTTQQSMIPAPTTHVGDLSLYDTSHNTSHSTSTANRSTSTTPGPYEAPMSQSRSQIVGHSNMQIPTSHSHMQNHQMGVNATGYDPRFSIQNYPVDPMMYKQ

**Ds ID68376**

MLISTTPGPVEVSISSRGHRTMPESSSSSTPAVKRACDACHRRKVRCIGDGTRPCKNCTSAGLTCTYNAIPQKKGPKGSRAKVISELRETQRQSQLAAKRNGWDLDSPAHSPADLKKAGLLSVEMMTACVDYFFANLYPTQPILHRQKVGEVIGQMETSIEAYCLVVSLCAYMMIQPNMVLPAEAFDGLEIPPQPSFQLGHTLLQEALRVRKGHNYIENPSIWSVITSFFFFGSYFCLDRHNTAWFHLREATTLAQIMGMHEEATYRTADTIESSRRRRLYWLLFVTERAYA

**En KHJ34074.1**

MSTSIKRACDACHRRKVKCDGINPCRNCYSAKLCCTYHAIPQKKGPKGSRAKVISELRENQRQSNLSSKVTNQANVINSPPYSPTLAPTQVLLSTEIIKECIDFFFTNMYPSLPILHRGRLEQQILFADQNIDTYCLLTSLCAFMMIQPGIKIAGNILERDCLPGTNLVSGNLLIQETLRIRRGYDYLEAPTLNSLVTSFFLFGCYFGLDLHNKAWFHLREATTLAHMNGLQKEETYLKFDAIDSSRRRRLFWLLFVTERAYAFQRNRPLSLQATISSPTQNDDPSDPQACHLNGFIDLVNLFRPFDEALVTLWNKTRTECSQNYLAGLQKDLLNALPSHLNSTETQNADLRTSQQWLRTMVWQLSMQNGCLSSTNSDPSMTFQYPVDISRDLVSLTSQFSPKSMDVHGIGFIEKLFDIACSLTDVLSLLPSTLDSLSFGPRDYLHDIIGLLAVLRHGDHQFLPLLLAKVNDVLPRLVNPMMRTIPDSSTDTNSNVCHEVGIFDDPGNATGIGITRPFLGVASRDSTNNGVVSKIEPNEFDPSNLIHGYPIFNHHSPQHQQHINEVISPILPSIECTKINTASSSCNEHGAAKSYSVSVQSPIQYSYSFPDIVNTTITTTPTTSTPVTTTNSFGNESKQKDFKAEFNHAIENPRRLPIRSCSGSSAPCVNSHGFSAPIPRSVLEQTYSLHRSQSFSCAEM

**Fg XP_382259.1**

MAGTIQKRACDGCHRRKVKCDSNSPCRNCNTANIQCTYNAIPQKKGPKGSRAKVISELRENQRLTSLPVKVQARMSGVPTESIQNHNVTNGLLTAEFAKSCLNFFFDNLYSKLPILDRDVIESQLSCMERDTEIYCLFTSLCAFMMLQPGMGFPTADYNLQMEPGANIAASNILVEECLRVRKGTEHWENPSHYVLATNYFIFAFYYSQQSHASAWYYLREATTMIQMSDQNNEGKYLQDMESIRRRRLYWLLFNAERAYAIQRSYPLTLQATVHTGNYGEDPADNLNPSIQNNFFNMCSVFQSIDDNFLSTWRHPCCRLDNQIIASLDAQLLKTVPTYPPNAALQAVQGWLKTVAWQTSGDTQENVAYRMSQFVSGFPNPAMLMNSGLIAKLLQVTSDLICYLVNAPQSRNPTTQGPERYLQTLLRICLAIQNNHYQFVPLLLSKVNEYLPRIVDPMLLNPPENVGYGMSVDIFDGFGNAGMAQMPMGDYNPGLPMNSYEPKYEDMGGNSPDSLPHSHHSHHSNGSPPGSQMGNEMPQNFVSSPGTVMSPGMDYTPSMGGFSISDMVMSPLGGAAPPNGINMQRQQQQQLNNQPQMQGMSNQGIGAPPMNQNIGSLYSGVRQPSRQSSFNMQSQTPLSAMGSMSDAMDFNTLPPR

**Lm XP_003838593.1**

MATNPTTTTPVKRACDSCHRRKVKCIGEGTAPCKNCVSAGLACTYNAVPQKKGPKGSRAKVLSELRENQRNAQLAAGYPSELGFDGRALTASFARTPGLLAPGLVESCIEYFFAHVYPSEPLLHRQRAQETAMNMDRSTESYCVIVALCAYVMIKANMKVSPTMLPRPEMAQMSNVSFGHILLEESVRVRQGYDFRENPTHLTVLTSYFYSGCYFGLGRENTAWAYLRDATTQAHILGMHDEDTYKHDPMDISRKRVLYWLLFIAERNFALHKHRPISLYPTIHPPTLDEASSDRQFASGLELMINMYKIIDDTFINLWNRVHTHANPAWIAQLQTQLAEAVPAYLDCNEAQSVEIRVTQHWLRAQAWQLCVTQGLVSSVTSDSPLTFKYPIEIARDLLTATHQFSQQSMEVHGAGLIEKLFDVACCLTDVVAVTSFSPDAFALGPRDYVSRFLTLISTLRGGQSRYLPLLLAKLSEVLPNLPLPRSLNLPQGVSTSSIGLSGTGSPTVPSNVGDDFSAMGQGTSPSYPSNDLIRRLAAQTGAQLPFNPPQHSSYPAPTSHVEDLSLYDTSHSTTHSSGSVPRSTSATPGPYEPSMSQSRTQIVGHSTMQIPTSHPQHNHMQSHHVEESAGTAVGAIGWVLHKGNGVEGCKLPDSPAQ

**Mj XP_007921440.1**

MPDSSSSSAAPSTTAKRACDACHRRKVKCIGDGTRPCKNCTSAGLTCTYNAIPQKKGPKGSRAKVISELRETQRQSQLAAKRHGFDLDSPPHSPAHLRQAGLLSVKMMTTCLDYFFANLYPTQPILHRQKVGEVIGQMEQNVEAYCLVVSLCAYMMIQPNMVIPPEAFDGLEIQPQPSLQLGHTLLQEALRVRRGYNYVENPSIWSVITSFFFFGSYFCLDRQNTAWFHLREATTLAQIMGMHDESSYPTNDLIENSRRRRLYWLLFVTERAYALQQHKPLTLHATINLPTLDEDPAETVELNGFIHLVHLFRPFDDTFVGLWNKARVGCTTEWLARLQQQLTDALPQYLQCTESQAVDLRCSQQWLRTMVWQLSISHGFLSSAAADNAMSFKYPIEISRDLVHSASQFSQQSMEVHGVGLIEKLFDVACTLTDVMSVIPQDQYTFEFGPRDYLNQLMTLISTLRGGQQRYMPLLMQKINDSMPNTGYALPAIPPSLRSEEVYDGSHSSAPTSHDSTPFGSPLSAGAQPFGYGEFSVTSAIQYPRTTEAGPMSNEIDYITAESHNLAGRQTEFETGG

**Mo XP_003710180.1**

MTTAVKRACDACHRRKVKCDGINPCRNCSSAQLSCTYNAIPQKKGPKGSRAKVISELRETQRQTSLATKVHNRLNGAPSPPASPAPAPSRGLLTKEMVSACIEFYFANMFSIIPILNRQQVEHDLGVWETDPETYSLMTSLCAFMMLQPGMSMPGGDPYGLDAQPGASIVSARLLMEETSRVRRLYDHLEKPSLNSVCTSYFLFACHYGLEEHDRAWYHLRDALTVTLMFGMNKEESYLTVDNATASRWRRLYWLLYMTERSYAIQRGRPLSLQATVNLPTLGDGPMDPFGTQLSSFLLLITIFRQFDDQFTNLWCKSRNNCSSSYINTLHKNFNEVLPHYLNNVDPQLADARNNQAWLKNLIWQLSMAKTQNVSDTTVQYPVDLQRELVAMTGAFSTQNTELINAGFIEKLCETSSSLIDVLTVQPATQNPFSPFNPRETLQQIFNLVGVIRKDESRFLPLLLCKMHEVIPRLISPMLQHMPDNMCSVDIFDGFGNAGMGQPSTMQMDDFDYRFPEPSIKTEPGTSSGASVPSVTDMHSPFSSSPPVISPQVEYPQGITQDFNPISDMMSPVGPGQQLNGPGGMGSHQTQHRMPQPPPQQHHQYTPTSAHNHPPKPTIQTMGHNMSTHHQNSLHGGINQPSNIPGQHPLPQQPLTSAAVRGPQPFSPAGAMNHSIMPGGPAMLMRPQPPQRSNSFAMPPPSNNTQLRTVGDFQTLQRTNSDMSVLTNSMGVSMGGGGGGMMGTEIDFNTLSR

**Ptt XP_003295651.1**

MASNPTTTAPVKRACDRKVKCIGEGTAPCKNCVSAGLACTYNAIPQKKGPKGSRAKVLSELRETQRNAQLAAGFPTDLGYDGRSLPSTFARTQGLLPPALVESCIEYFFNNVYPTEPVLHRQRAQEAAVGMDRSTEAYCMIVALCAYVMIQGNHKPHVNVLPRQEMAHMSNVGIGHVLLEESVRVRQGYDHRENPTHMSVLTSYFYHGCYFGLARENTAWTYLREATTQAQLLGMHDEETYKHDPLDVSRKRVLYWLLFIAERTYALHKRRPITLHPTIHTPSLDEVPSDRPIAVGLELMINMFKIIDDNFINLWNRVNNTHASAAWIAQVQTQLSEAVPAYFECTEAQEVQIRITQQWLRSQAWQLSVSQGLVSSVTNDPPLTFKYPIEISRDLLTISHQFSQQALEVHGVGLIEKLFDIACCLTDVVACTTFSPDQFALGPRDYVSRFLTLISTLRGGQSRYLPLLLSKLNEVLPNLPLPRSLNLPQTVSTSTLGLNASGSGTLPSNVADDFSASPASSSPSYPTNDLIRRIAAQTGAQLPFNTTQQSMISAPTTHVGDLSLYDTSQQSTTHSSSSGPRSNSTTPGPYETPLSQSRSQIVGHSAMQLPTSHGHMQSHHMGVNPTAYDPRFSIQNYPVDPMMYKH

**Sb ESZ93518.1**

MSSNSVKRACDACHRRKVKCDGINPCRNCFSTQLSCTYHAIPQKKGPKGSRAKVISELRNNQQSSLSSKTANRMNGVPSPPCSPTLAPTPGLLTTEMVKECTEFFFANMYPTMPILHRERLEQQAMYVDQNVDTYCLLTSMCAFMMIQPGMGIPGDPLGLDNLPGANIVSGTLLMEETLKVRKSYDYCESPTHNSLVTSFFLFACYYGLDLHNKAWFHLREATTLAHTLEMVKEETYHQFDIIESSRRRRLYWLLFVTERSYALQRHRPLSLQSTINLPSQNDDPTDPQNHHLHGFIHLVNVYRPFDDQFVALWNKTRTDCSPAYLSSLQKQLSEALPIYLNSTENQTADLRTSQQWLRTMVWQLSIQNGCLSSNCDDPSMSFQYPVDIARELAQMTSQFSHQSMEVHGVGLIEKLFDVACSLTDVLSLLPAKPDPFAMGPRDYLHQFMTMLSILRNGDHRFLPLLLNKVHDVLPGLTNPMLQNVPENTQMCADVDLFDGFGNTGISIPPQNYNSMATSGPDEYKMGHTNGGLAFDKRIEELSSPSVTGSMSAETTPFTSPPIMNPGMEYQALDYPGFHDLTGHNHGMDFKREYNVENRESSFGVSSPGGMSEMSNGVVSVNGNGNGIGIGGRQPPIRQGSGSSYGMPLPRSIPEFNHGHFSRSSTGGEDMGVGGNGVDLGHHGYR

**St XP_008031030.1**

MASNPTSTAPVKRACDSCHRRKVKCIGEGTAPCKNCVSAGLACTYNAIPQKKGPKGSRAKVLSELRETQRNAQLAAGFPTDLGFDGRSLPATFARTQGLLPPALVESCIEYFFNHVYPSEPVLHRQRAQEAAVGMDRSTESYCMIVALCAYVMIQANHKPPVSVLPRPEMAHMSNVGVGHILLEESVRVRQGYDHRENPTHMSVLTSWFYSGCYFGLARENTAWTYLREATTQAQLLGMHDEETYKHDPLDVSRKRVLYWLLFIAERTYALHKHRPITLHPTIHPPSLDEVPTDRPIAVGLELMINMFKIIDDTFINIWNRVHNTHVSAAWITQVQTQLSEAVPAYFECSEAQEVQIRITQQWLRSQTWQLSVCQGLVSSVSNDMPLTFKYPIEIARDLLTISHQFSQQSMEIHGVGLIEKLFDIACCLTDVVACTSFSPDAFALGPRDYVSRFLTLISTLRGGQSRYLPLLLAKLSEVLPNLPLPRSLNLPQTVPASTLGLNATGSGTLPSNVAEDFSASPASSSPSYHSNDLIRRLAAQTGAQLPFNTTQQSMIPAPTTHVGDLSLYDSSHSTSHSTSTANRSTSTTPGPYEAPMSQPRSQIVGHNAMQIPTSHSHMQTHQMGVNATGYDPRFSMQNYPVDPMMYKQ

**Zt XP_003857279.1**

MLISTLSGPVEVPSVSPHQRPTSRDMADTVLPVPSSVKRACDGCHRRKVKCIGDGTRPCKNCTSAGLTCTYNAIPQKKGPKGSRAKVISELRETQRQSQLVAKRHGLDFEHETHHAQAYPRKPGLLSMDMITTCVDYYFANIYPTQPILHRQKVGETIGQMDTNIEAYCLVLSLCAYMMIQPNMVIPHGAFEGLDILPQPSLQVGHLLLQEALRMRKEYNYIETPTVWSVITSFFFFGSYFCLDRQNTAWFHLREATTLAQVMGMHEEASYQNPDVVESSRRRRLYWLLFVTERAYALQQHKPLTLHATINLPTLDEDPAETVELNGFIHLVRLFRPFDDTFVGLWNKARVGCTTEWLARLQQQLSDALPTYLQCTETQAVDLRCSQQWLRTMVWQLSISHGFLSSAAADNAMSFKFPIEVSRDLVQSASQFSQQSMEVHGIGLIEKLFDVACTLTDVMSVIPQDQYTFELGPRDYLNQLTSLISSLRGGQQRYLPLLMQKISDAVPDHQTPAFTIPAIPRSIRSEEIYDGSHSSAPNSGESTPAFGSPLGSPLSVGVNPYNSYPDMGVTSALQYGPPPPAATYSMTSAPTTVPGPQAMYGMPNPAVTRGPVPKYDSAG
