# Supplementary figures and images for: A functionally conserved Zn2Cys6 binuclear cluster transcription factor class regulates necrotrophic effector gene expression and host‐specific virulence of two major Pleosporales fungal pathogens of wheat
Source: Mol Plant Pathol. 2017 Jan 24;18(3):420–34. doi: 10.1111/mpp.12511 (PMC6638278; doi:10.1111/mpp.12511)

**
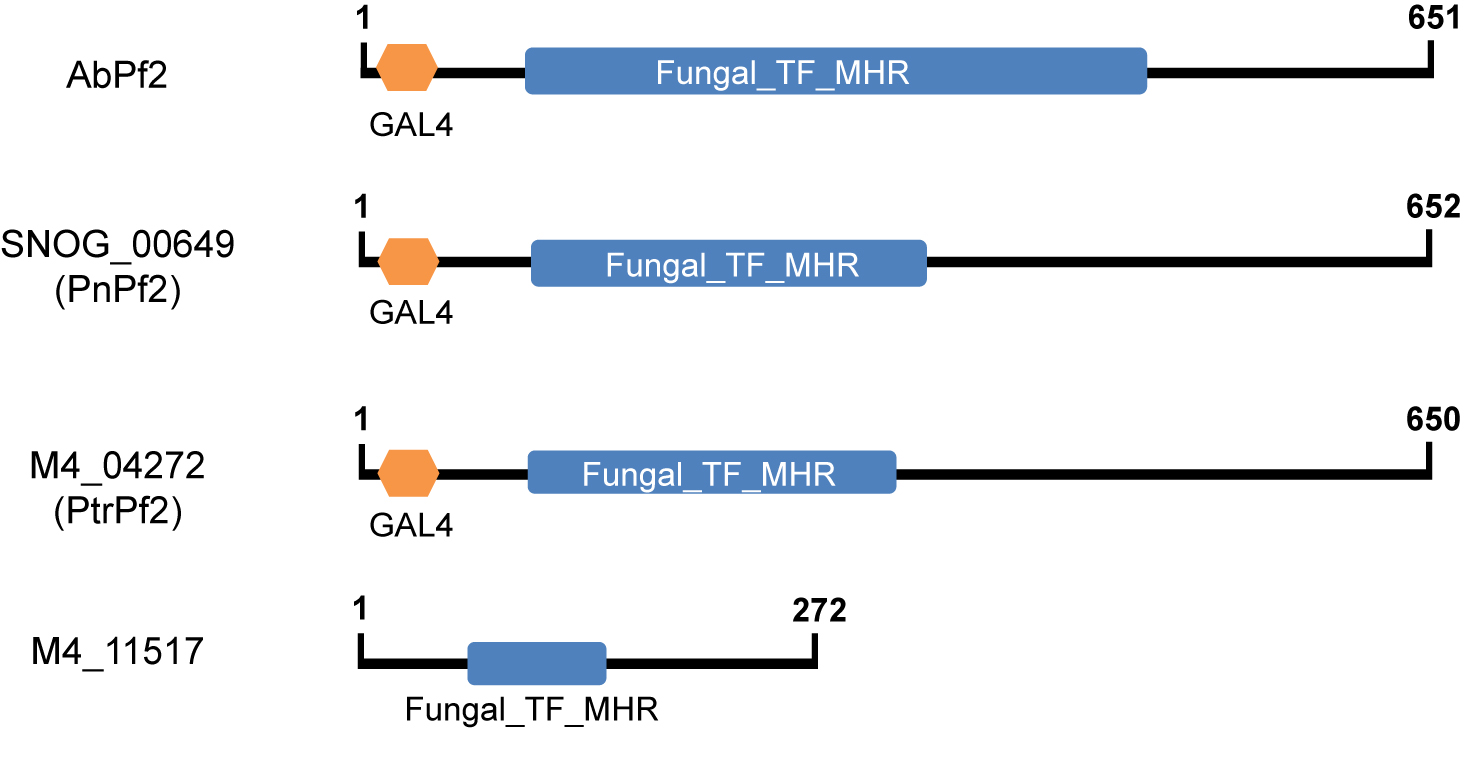
**

Supplement: Supplementary file 4 — Fig. S1 Scaled schematics of the CD‐Blast conserved domain structure for SNOG_00649 (PnPf2), M4_04272 (PtrPf2), M4_11517 and AbPf2. Amino acid numbers are shown. The GAL4‐like Zn2Cys6 binuclear cluster domain is indicated in orange and the fungal transcription factor regulatory middle homology region is indicated in blue. [file MPP-18-420-s004.docx]

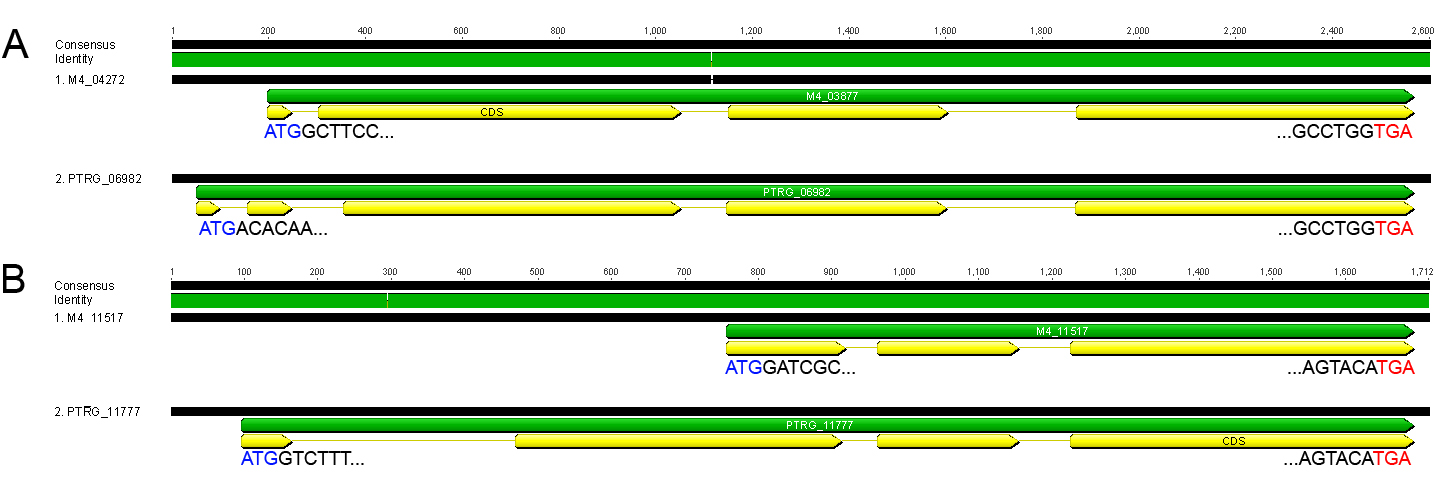

Supplement: Supplementary file 5 — Fig. S2 Nucleotide alignment between M4_04272 and PTRG_06982 (A) and M4_11517 and PTRG_11777 (B). Start (blue) and stop (red) codons are indicated. [file MPP-18-420-s005.docx]

*
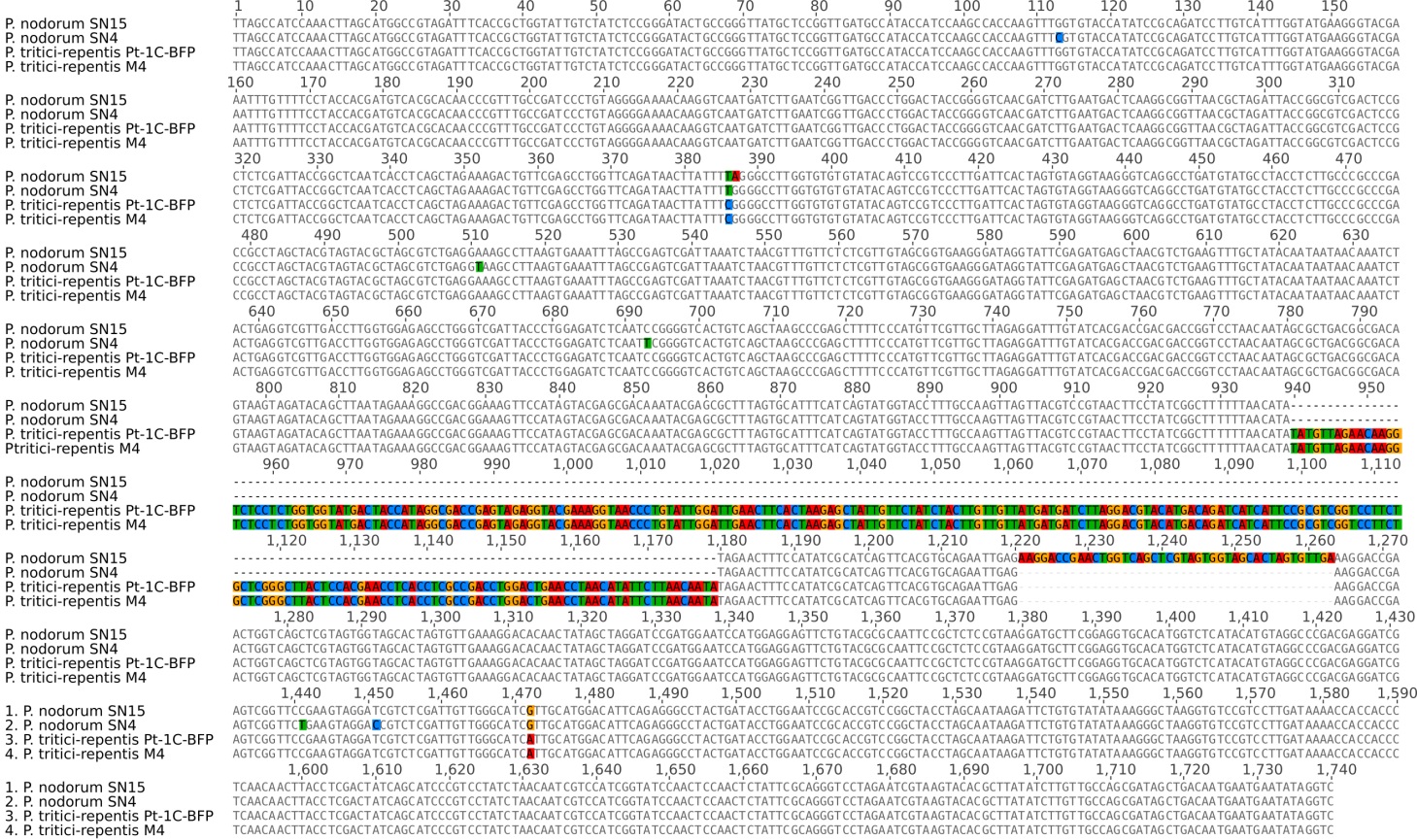
*

Supplement: Supplementary file 6 — Fig. S3 Nucleotide alignment of the putative promoter region of ToxA (yellow) from Parastagonospora nodorum (SN15 and SN4) and Pyrenophora tritici‐repentis (M4 and Pt‐1C‐BFP). [file MPP-18-420-s006.docx]

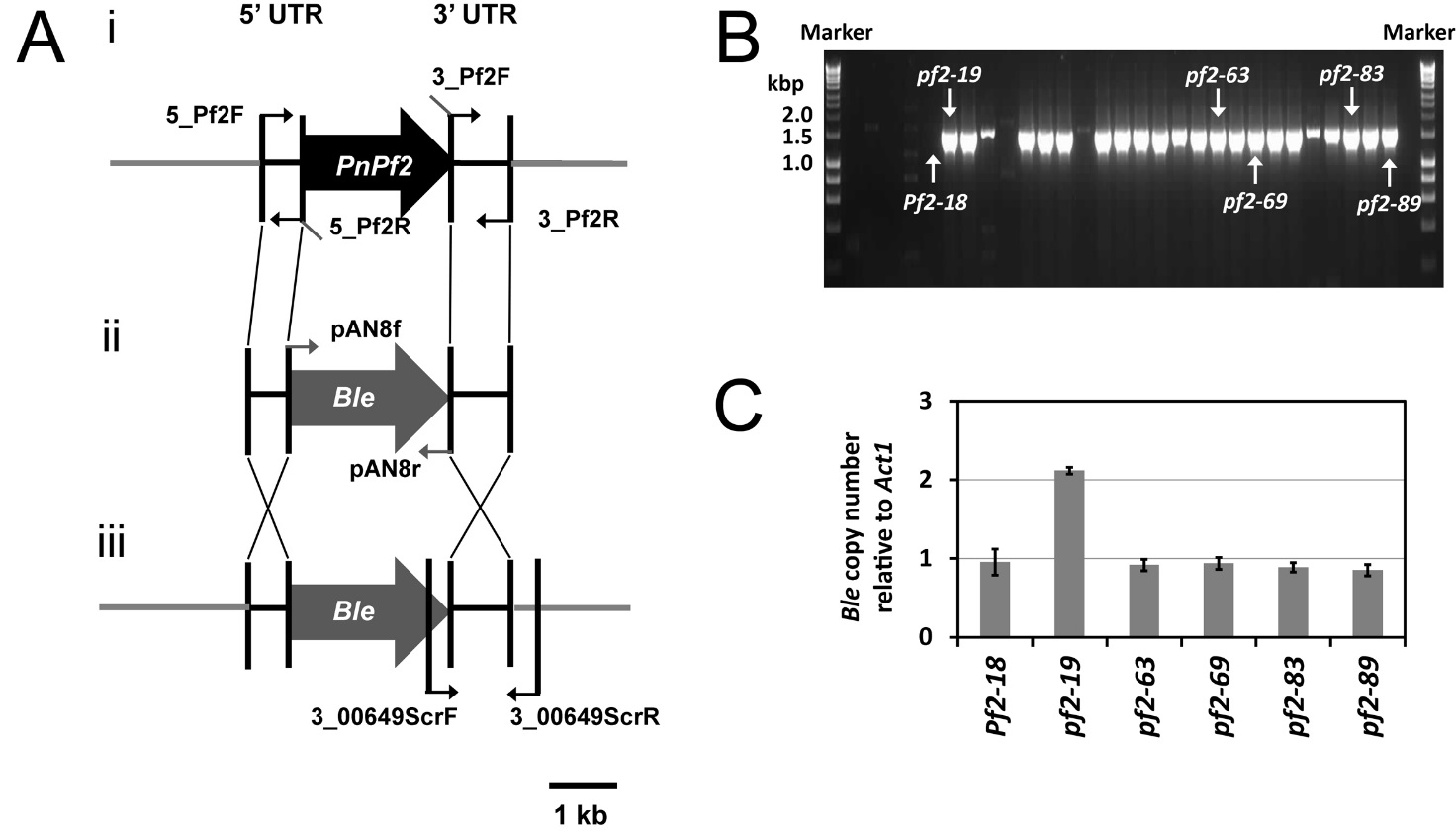

Supplement: Supplementary file 7 — Fig. S4 Construction of the PnPf2 knockout vector. (A) The 5′ and 3′ untranslated regions (UTRs) of PnPf2 were polymerase chain reaction (PCR) amplified (i) and fused to Ble to give the PnPf2 knockout vector (ii). (iii) The vector was transformed into SN15 to facilitate gene knockout via homologous recombination of the 5′ and 3′ flanks. (B) Five knockout mutants (pf2–) and two ectopic strains (Pf2–) were selected from PCR screening using 3_00649ScrF/R for insert copy number determination using quantitative PCR. (C) All possess single‐copy integration, except for pf2‐19. Consequently, pf2‐63, pf2‐69 and Pf2‐18 (Ect) were selected for further studies. [file MPP-18-420-s007.docx]

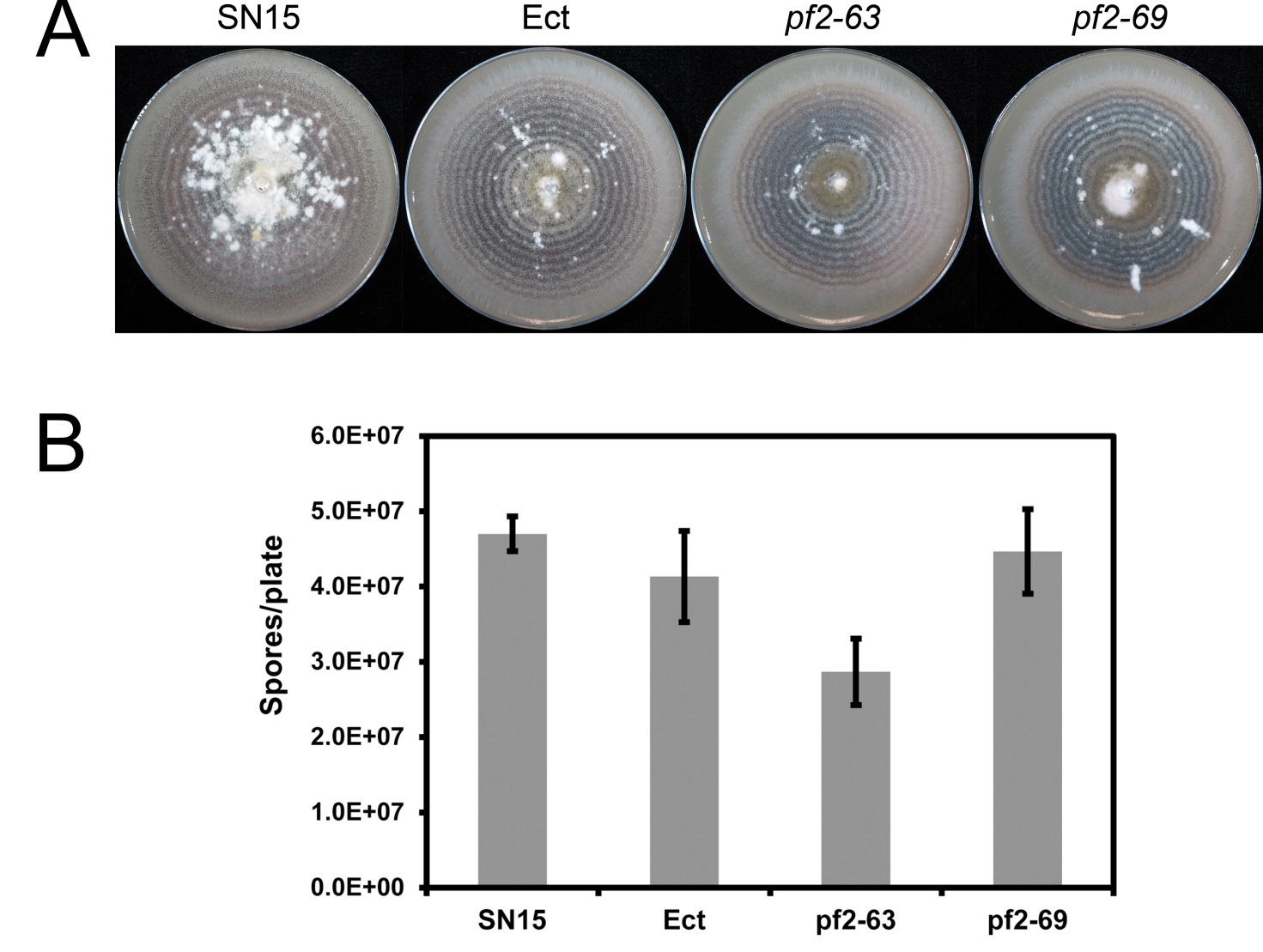

Supplement: Supplementary file 8 — Fig. S5 Assessment of colony morphology (A) and pycnidiospore production (B) on 2‐week‐old Petri dish fungal cultures grown on V8‐PDA. Error bars are shown as standard error of the mean. The experiment was performed in biological replicates (n = 3). [file MPP-18-420-s008.docx]

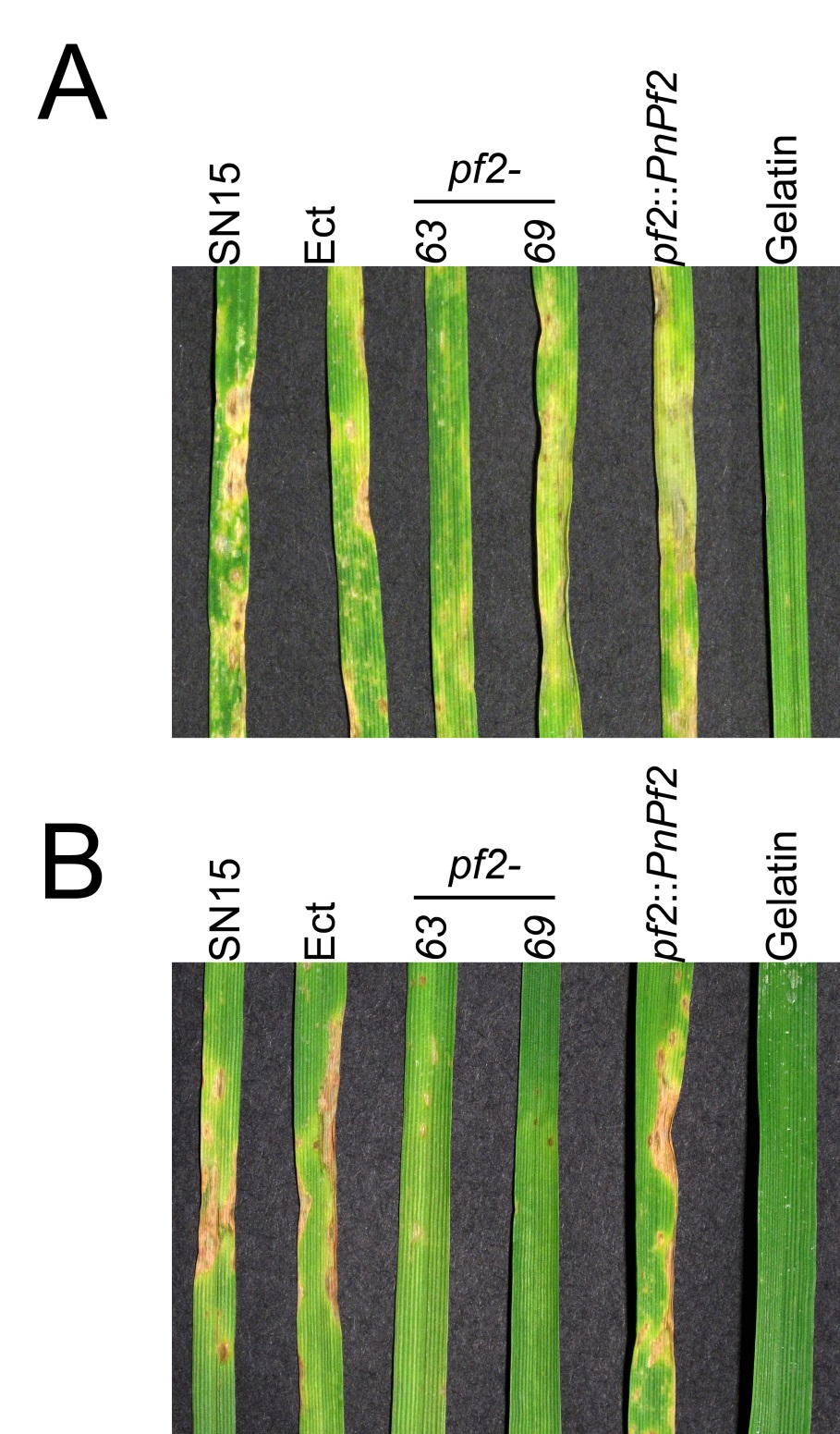

Supplement: Supplementary file 9 — Fig. S6 Whole‐plant virulence assay of Parastagonospora nodorum strains on wheat cv. Chinese Spring (tsn1, Snn1, snn3) (A) and Wyalkatchem (tsn1, snn1, Snn3) (B). [file MPP-18-420-s009.docx]

**
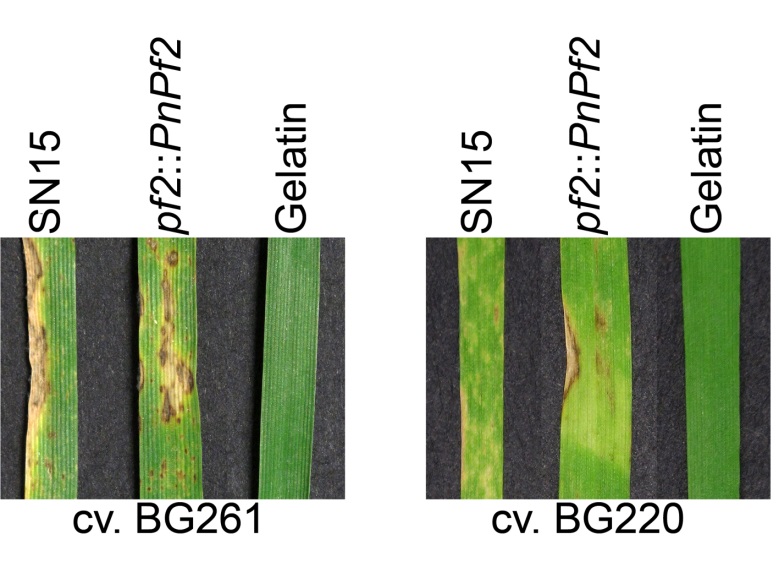
**

Supplement: Supplementary file 10 — Fig. S7 Genetic complementation of pf2‐69 restores virulence on BG261 and BG220. [file MPP-18-420-s010.docx]

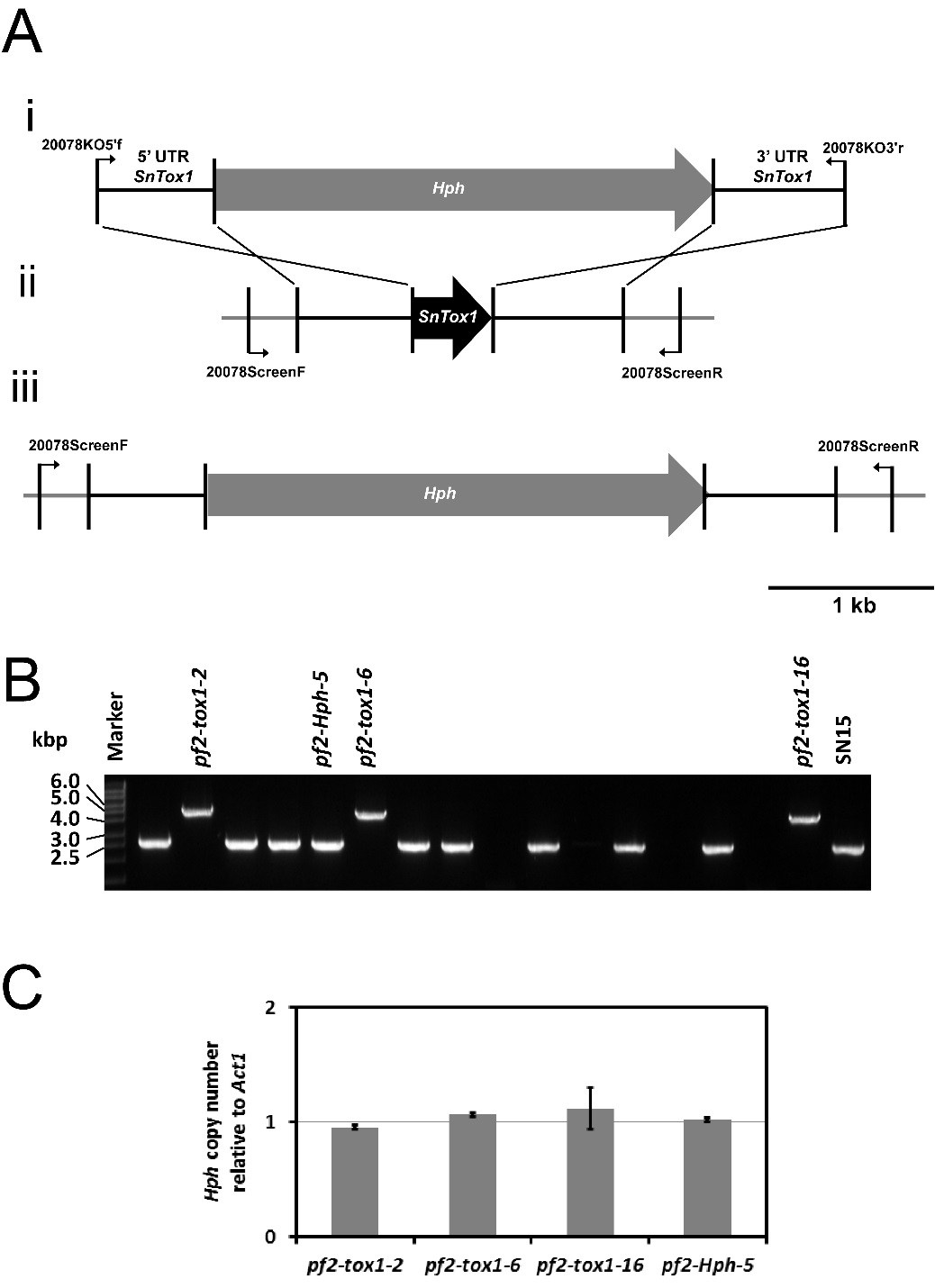

Supplement: Supplementary file 11 — Fig. S8 SnTox1 deletion in pf2‐69. (A) The SnTox1‐Hph gene knockout cassette was polymerase chain reaction (PCR) amplified from Parastagonospora nodorum tox1‐6 (i). This was used to delete SnTox1 in pf2‐69 (ii), resulting in mutants that lacked PnPf2 and SnTox1 (iii). (B) PCR using the primer pair 20078ScreenF/R was used to screen for SnTox1 deletion in all transformants. Mutants deleted in SnTox1 produced a 4.9‐kb PCR amplicon. PCR amplification of ectopic transformants resulted in a 2.6‐kb band. (C) One ectopic and three tox1 mutants were analysed for insert copy number using quantitative PCR. All strains possess single‐copy integration. [file MPP-18-420-s011.docx]

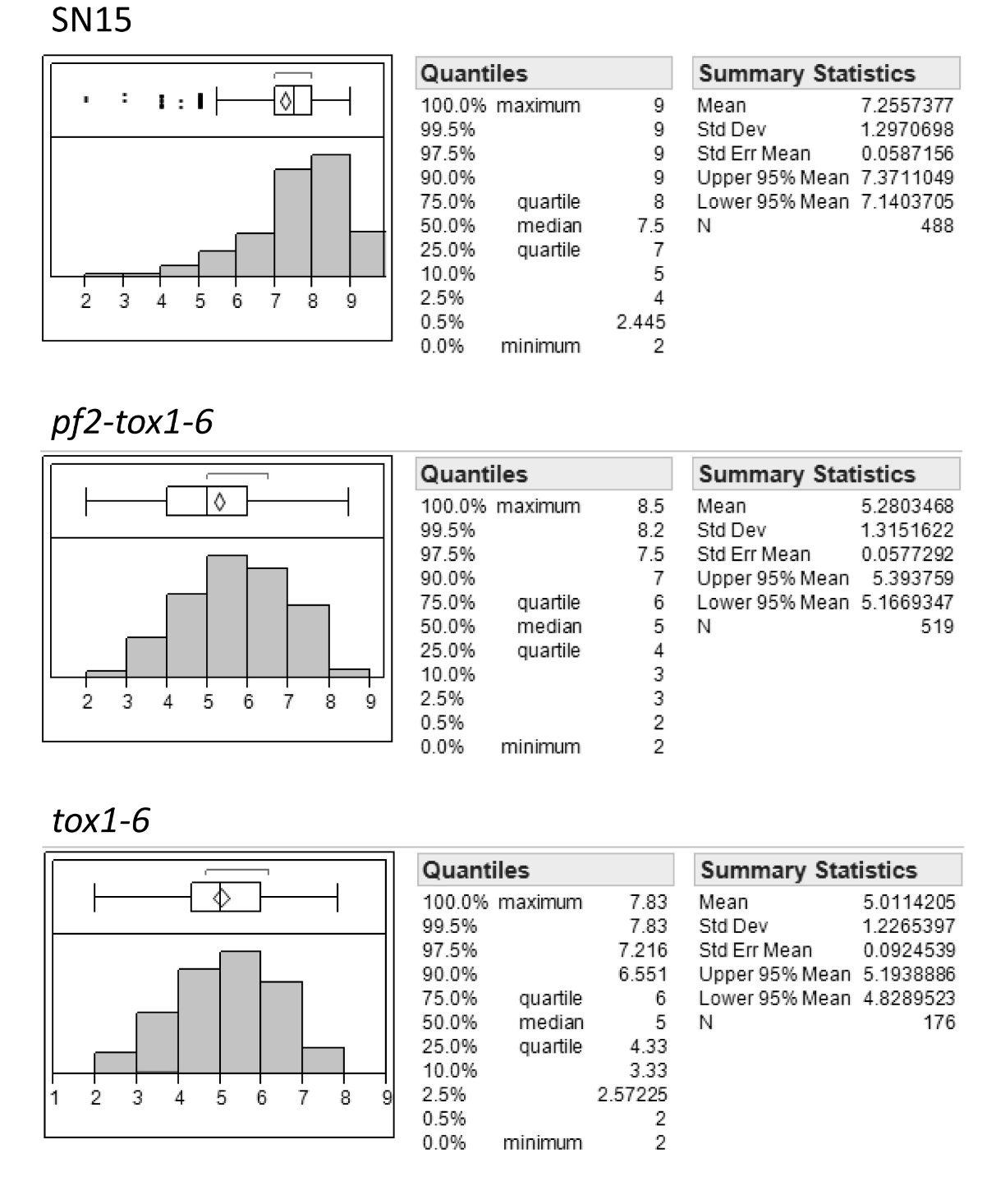

Supplement: Supplementary file 12 — Fig. S9 The distribution of SN15, pf2‐tox1‐6 and tox1‐6 whole‐plant spray disease severity scores on the Calingiri × Wyalkatchem DH population at the seedling stage. Data from SN15 and tox1‐6 were derived from Phan et al. (2016). [file MPP-18-420-s012.docx]

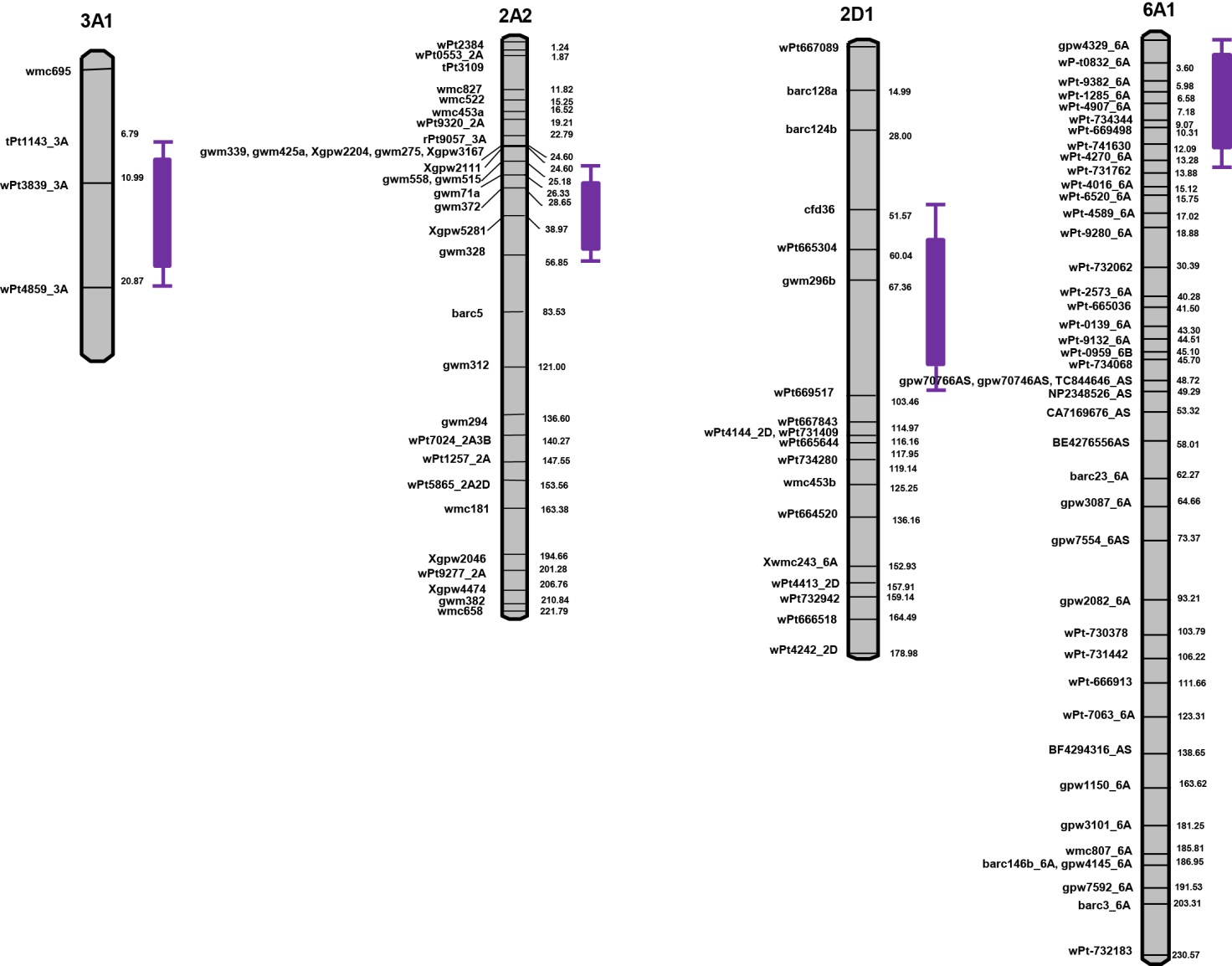

Supplement: Supplementary file 13 — Fig. S10 A genetic map of chromosomes with genetic markers on the right and the centimorgan (cM) distances between loci on the left. Quantitative trait loci (QTLs) associated with pf2‐tox1‐6 infection are indicated in purple. [file MPP-18-420-s013.docx]

**
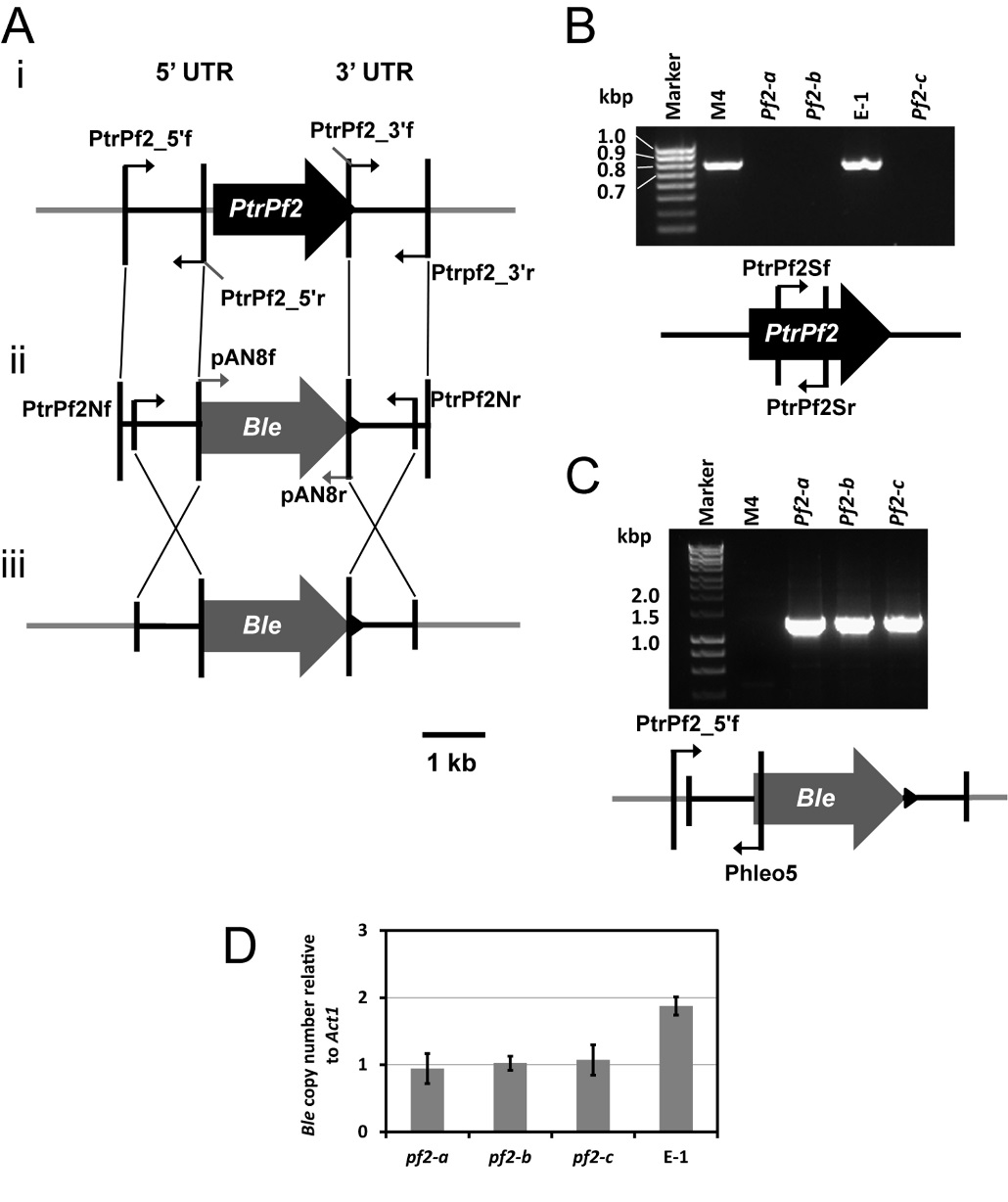
**

Supplement: Supplementary file 14 — Fig. S11 Construction of the PtrPf2 knockout vector. (A) The 5′ and 3′ untranslated region (UTR) of PtrPf2 was polymerase chain reaction (PCR) amplified (i) and fused to Ble to give the PtrPf2 knockout vector (ii). This was amplified with the nested primer pair PtrPf2Nf/PtrPf2Nr and transformed into the Pyrenophora tritici‐repentis M4 wild‐type to facilitate gene knockout (iii). (B) PtrPf2‐specific amplification using the primer pair PtrPf2Sf and PtrPf2Sr identified three knockout (pf2–) and one ectopic (E‐1) mutant. (C) Gene disruption of the PtrPf2 locus was confirmed using the primer pair PtrPf2_5′f Phleo5 which amplifies a 1.4‐kb fragment in strains that carry the appropriate gene deletion. (D) Transformants were analysed for insert copy number using quantitative PCR. All strains possess single‐copy integration except for E‐1. Pyrenophora tritici‐repentis is very difficult to transform. Consequently, we were only able to identify one ectopic mutant. However, double integration did not reduce the level of fitness in the E‐1 strain. [file MPP-18-420-s014.docx]

**
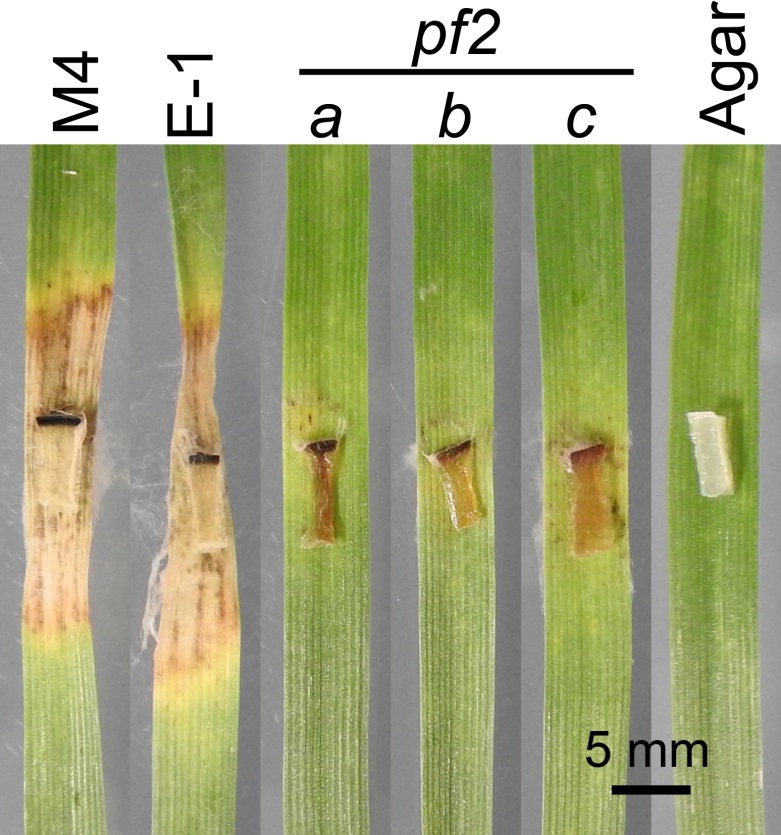
**

Supplement: Supplementary file 15 — Fig. S12 Virulence of Pyrenophora tritici‐repentis M4 wild‐type, E‐1 and PtrPf2 deletion mutants on wheat cv. Yitpi (Tsn1). [file MPP-18-420-s015.docx]

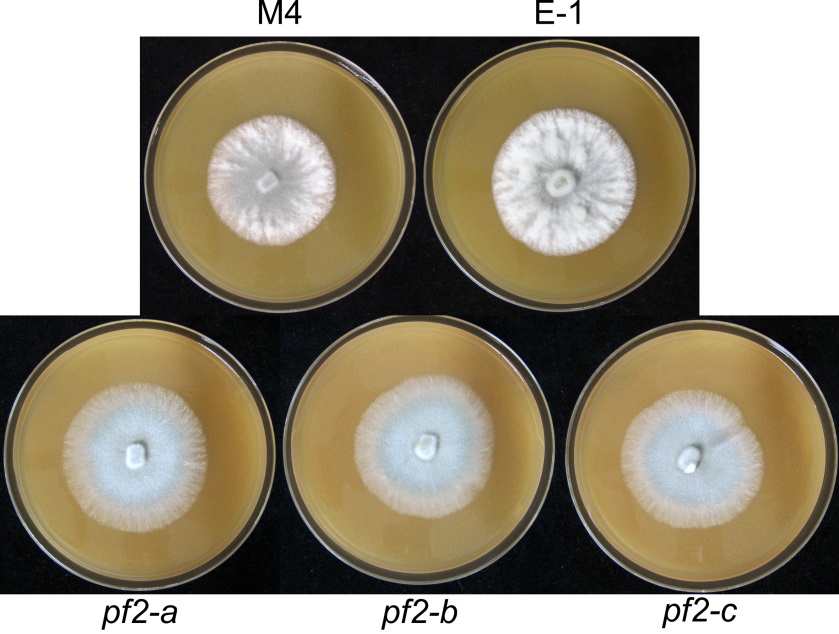

Supplement: Supplementary file 16 — Fig. S13. Colony morphology of the P. tritici‐repentis M4 wild‐type, E‐1 ectopic and PtrPf2 deletion strains on V8‐PDA. [file MPP-18-420-s016.docx]

**
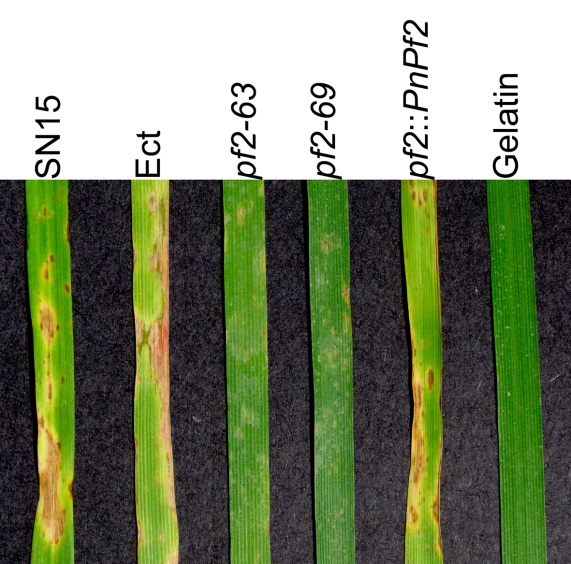
**

Supplement: Supplementary file 17 — Fig. S14 Whole‐plant virulence assay of Parastagonospora nodorum strains on wheat cv. BG223. [file MPP-18-420-s017.docx]

**
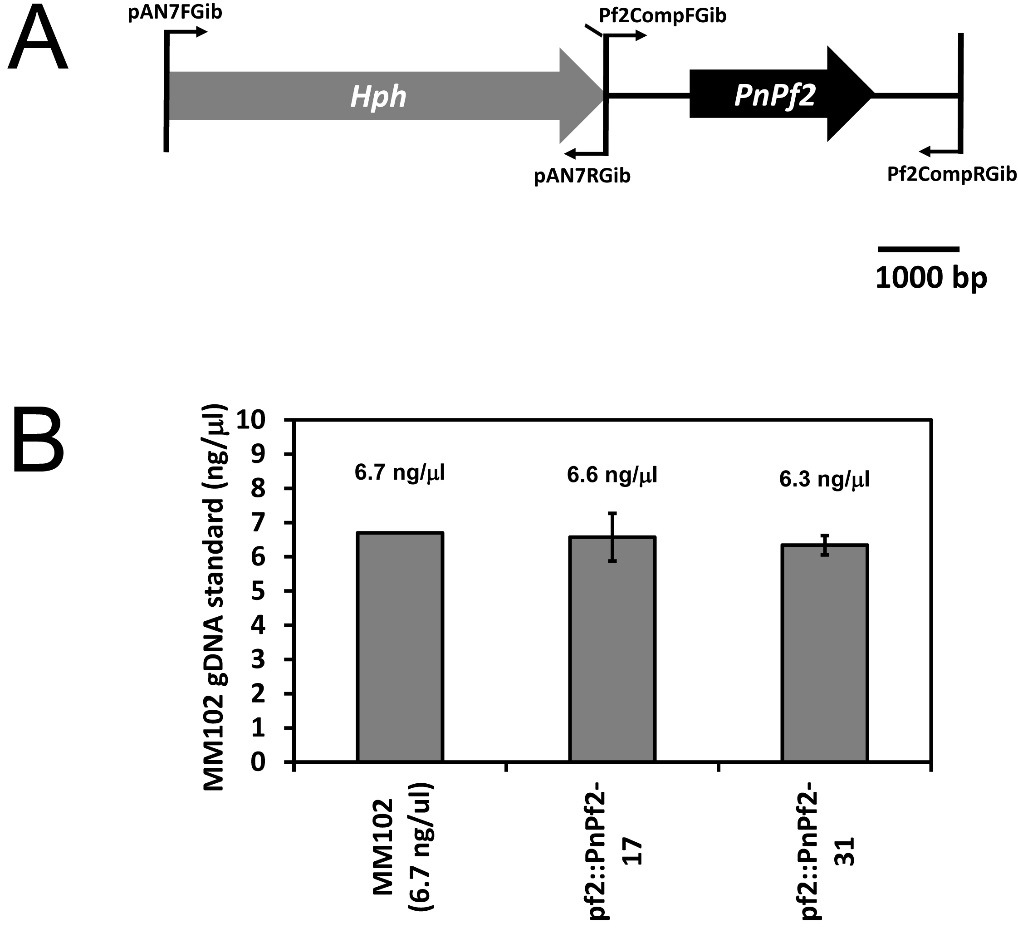
**

Supplement: Supplementary file 18 — Fig. S15 Genetic complementation of Parastagonospora nodorum pf2‐69. (a) Fusion polymerase chain reaction (PCR) was used to construct the PnPf2‐Hph gene complementation vector. (b) PnPf2‐Hph insert copy number determination using quantitative real‐time PCR. Biological triplicates were used in the copy number assay. Error bars are shown as the standard error of the mean. [file MPP-18-420-s018.docx]
